# Extensive Changes in Transcriptomic “Fingerprints” and Immunological Cells in the Large Organs of Patients Dying of Acute Septic Shock and Multiple Organ Failure Caused by *Neisseria meningitidis*

**DOI:** 10.3389/fcimb.2020.00042

**Published:** 2020-02-19

**Authors:** Berit Sletbakk Brusletto, Else Marit Løberg, Bernt Christian Hellerud, Ingeborg Løstegaard Goverud, Jens Petter Berg, Ole Kristoffer Olstad, Unni Gopinathan, Petter Brandtzaeg, Reidun Øvstebø

**Affiliations:** ^1^Department of Medical Biochemistry, Oslo University Hospital, Oslo, Norway; ^2^Institute of Clinical Medicine, University of Oslo, Oslo, Norway; ^3^Department of Pathology, Oslo University Hospital, Oslo, Norway; ^4^Institute of Immunology, Oslo University Hospital, Oslo, Norway; ^5^Department of Pediatrics, Oslo University Hospital, Oslo, Norway

**Keywords:** FFPE, IHC, MOF, *Neisseria meningitidis*, septic shock, transcriptional profiles

## Abstract

**Background:** Patients developing meningococcal septic shock reveal levels of *Neisseria meningitidis* (10^6^-10^8^/mL) and endotoxin (10^1^-10^3^ EU/mL) in the circulation and organs, leading to acute cardiovascular, pulmonary and renal failure, coagulopathy and a high case fatality rate within 24 h.

**Objective:** To investigate transcriptional profiles in heart, lungs, kidneys, liver, and spleen and immunostain key inflammatory cells and proteins in post mortem formalin-fixed, paraffin-embedded (FFPE) tissue samples from meningococcal septic shock patients.

**Patients and Methods:** Total RNA was isolated from FFPE and fresh frozen (FF) tissue samples from five patients and two controls (acute non-infectious death). Differential expression of genes was detected using Affymetrix microarray analysis. Lung and heart tissue samples were immunostained for T-and B cells, macrophages, neutrophils and the inflammatory markers PAI-1 and MCP-1. Inflammatory mediators were quantified in lysates from FF tissues.

**Results:** The transcriptional profiles showed a complex pattern of protein-coding and non-coding RNAs with significant regulation of pathways associated with organismal death, cell death and survival, leukocyte migration, cellular movement, proliferation of cells, cell-to-cell signaling, immune cell trafficking, and inflammatory responses in an organ-specific clustering manner. The canonical pathways including acute phase response-, EIF2-, TREM1-, IL-6-, HMBG1-, PPAR signaling, and LXR/RXR activation were associated with acute heart, pulmonary, and renal failure. Fewer genes were regulated in the liver and particularly in the spleen. The main upstream regulators were TNF, IL-1β, IL-6, RICTOR, miR-6739-3p, and CD3. Increased numbers of inflammatory cells (CD68+, MPO+, CD3+, and CD20+) were found in lungs and heart. PAI-1 inhibiting fibrinolysis and MCP-1 attracting leukocyte were found significantly present in the septic tissue samples compared to the controls.

**Conclusions:** FFPE tissue samples can be suitable for gene expression studies as well as immunostaining of specific cells or molecules. The most pronounced gene expression patterns were found in the organs with highest levels of *Neisseria meningitidis* DNA. Thousands of protein-coding and non-coding RNA transcripts were altered in lungs, heart and kidneys. We identified specific biomarker panels both protein-coding and non-coding RNA transcripts, which differed from organ to organ. Involvement of many genes and pathways add up and the combined effect induce organ failure.

## Introduction

*Neisseria meningitidis* is feared among health care personnel and lay people owing to its propensity to cause acute meningitis and fulminant septicemia, often in clusters of the population (van Deuren et al., 2000; Rosenstein et al., 2001; Stephens et al., 2007). The case fatality rate of systemic meningococcal disease (SMD) has remained fairly stable around 10% since the introduction of sulfa therapy in the 1930s and penicillin in the 1940s (Barquet et al., 1999; Brooks et al., 2006; Stanton et al., 2011; Stoof et al., 2015).

Septic shock is the principal cause of death in patients with SMD in industrialized countries (Gedde-Dahl et al., 1983; Halstensen et al., 1987; de Greeff et al., 2008; Brandtzaeg and Van Deuren, 2012). Persistent septic shock in SMD is a consequence of the rapid proliferation of *N. meningitidis* in the circulation and in the vasculature of various organs. This is reflected by very high levels of *N. meningitidis* (10^6^-10^8^) in blood and tissues (10^4^-10^9^) as measured by the number of meningococcal DNA molecules (NmDNA), levels of meningococcal lipopolysaccharides (LPS, endotoxin) in the range of 10^1^-10^3^ endotoxin units (EU)/ mL plasma and an accompanying immune response which is dose-related to the LPS levels in plasma (Brandtzaeg et al., 1989a; van Deuren and Brandtzaeg, 2000; Hackett et al., 2002; Brandtzaeg and Van Deuren, 2012).

The septic shock is characterized by a profound vasodilation, reduced ventricular contractility, vascular leakage reducing the circulation blood volume and a gradual development of inflammation-induced cardiac failure, manifested by increased left ventricle volume and decreasing ejection fraction (Boucek et al., 1984; Mercier et al., 1988; Hagmolen of ten Have et al., 2000; Thiru et al., 2000; Brandtzaeg, 2006; Martin et al., 2019). The septic cardiac failure and vasodilation have been associated with increased production of nitric oxide (NO) in endothelial cells, high levels of tumor necrosis factor (TNF), interleukin 1β (IL-1β), IL-6, vasoactive intestinal polypeptide and persistent high levels of anaphylatoxins C3a and C5a in plasma (Waage et al., 1989a; Baines et al., 1999; Pathan et al., 2004; Brandtzaeg, 2006; Kobsar et al., 2011).

Concomitantly, the coagulation system is triggered by multiple factors and leads to disseminated intravascular coagulation (DIC) and formation of thrombi and hemorrhages in different tissues and organs including the adrenals, lungs, kidneys, skin, and extremities (Ferguson and Chapman, 1948). Formation of thrombi in capillaries and larger vessels is the hallmark of meningococcal septic shock. The balance between coagulation and fibrinolysis is tilted toward coagulation. Monocytes and monocyte derived microvesicles carrying functional active tissue factor appear to initiate the process in plasma (Osterud and Flaegstad, 1983; Ovstebo et al., 2012, 2014; Hellum et al., 2014). Complement factor 5, which is massively activated to C5a and C5b in these patients, augments the coagulation cascade and thrombus formation (Brandtzaeg et al., 1989b; Ovstebo et al., 2014). The fibrinolytic system is severely impaired by plasminogen activator inhibitor 1 (PAI-1). The low levels of protein C and to a lesser extent antithrombin, are associated with purpura fulminance i.e., diffuse thrombotic lesions of the skin. The endothelial cells reveal concurrently reduced levels of thrombomoduline and protein C receptors. Adherence of leukocytes, primarily neutrophils, and meningococci to the endothelial cells may alter the surface and facilitate thrombus formation in the skin, various organs and peripheral extremities (Brandtzaeg et al., 1989c, 1990; Hazelzet et al., 1996; de Kleijn et al., 1998; Faust et al., 2001; Melican et al., 2013). Meningococcal septic shock is often described as overwhelming owing to rapid development of the symptoms, high levels of the bacterium in the circulation and large organs as well as endotoxin levels in plasma higher than measured in any other human infections. This clinical entity is clearly different from most cases of septic shock and multiple organ failure as observed in intensive care units in present day hospitals. However, studies of the gene regulation in lungs, heart, kidneys, liver and spleen of meningococcal infection may provide valuable information in our understanding of multiple organ failure induced by other organisms and in other clinical settings.

Previous studies have applied transcriptomic methods to investigate changes in the gene expression underlying the inflammatory response in meningococcal sepsis and other forms of sepsis (Maslove and Wong, 2014). These studies have primarily been conducted in whole blood or leukocyte fractions (Maslove and Wong, 2014). Studies of meningococcal sepsis in an *in vivo* porcine model and in patients suggest that the concentration of *N. meningitidis* may be significantly higher in the major organs as compared to blood (Hellerud et al., 2015; Brusletto et al., 2017). These findings suggest that the local inflammatory response in different organs contributes quantitatively to the organ dysfunction. Yet no studies have so far investigated changes in transcriptional profiles and the corresponding activation of signaling pathways in major organs affected by massive proliferation of *N. meningitidis*.

The primary aim of this study was to investigate gene expression changes in details induced in patients who died of meningococcal septic shock and to identify activation of signaling pathways that might explain the development of organ dysfunction in these patients. We used both paraffinized and fresh frozen tissues collected during routine autopsies in which the bacterial load in each organ previously had been determined (Hellerud et al., 2015; Brusletto et al., 2017). To our knowledge this has not been done before. A secondary aim was to visualize the immune reaction by immunohistochemistry and quantify various immune cells and specific molecules that might play crucial roles in the pathophysiology which develops in the heart and lungs in lethal meningococcal septic shock. We also compared results between paraffinized and fresh frozen tissues to generate knowledge about the influence of storage methods on human material.

## Materials and Methods

### Ethics Approval and Consent to Participate

The study was approved by the Regional Medical Ethical Committee of South East Norway (2011/1413C “Translational research, meningococcal disease” and 2011/753 “Studies of invasive meningococcal and pneumococcal disease”). The patients' samples were collected after informed consent from patient parents or relatives and according to the Helsinki declaration. The Director of Public Prosecutions approved the use of forensic material for this research.

### Clinical Definitions

Systemic meningococcal disease (SMD) was present if *N. meningitidis* was cultivated and or confirmed by polymerase chain reaction (PCR) in blood, cerebrospinal fluid (CSF) or by organ tissue examination (Brandtzaeg et al., 1989a; Ovstebo et al., 2004; Brusletto et al., 2017). Severe septic shock was defined as persistent hypotension because of bacterial infection, with a systolic blood pressure <90 mm Hg in adults (≥12 year) and <70 mmHg in children (<12 year), that required fluid therapy and treatment with vasoactive drugs (dopamine, epinephrine, norepinephrine) for at least 24 h or until death (Brandtzaeg et al., 1989a).

Multiple organ failure was defined as: (1) reduced pulmonary function requiring artificial ventilation to maintain an adequate arterial oxygenation and (2) renal failure as reduced creatinine clearance (<60 mL/minute per 1.73 m2 body surface) or pathologically elevated serum creatinine (related to age and collected within 12 hours after admission).

### Time Between Hospital Admission and Death

Patients 1–4 (Table 1) died within 270 min of hospital admission. Patient 5 was found dead in bed.

**Table 1:** Overview of patients, clinical data, storage age of tissue, and type of tissue.

**Patient no**	Neisserial DNA; copy number of *N. meningitidis/*mL LPS (LAL); EU/mL at admission to hospital ^*^not available	**Age of tissue at isolation time of DNA/RNA (years)**	**Type of storage methods**	**Type of organ tissue**	**Copies** ***N. meningitidis*** DNA/ug human DNA ^*^not available		**Type of organ tissue included in the study**	
					**FFPE**	**FF**	**FFPE**	**FF**
1 Patient with systemic meningococcal disease (SMD) with shock	2.8 × 10^8^ copies/mL (plasma) 2100 EU/mL (plasma) No spinal puncture was performed	11	FFPE	Lungs Heart Kidneys Liver Spleen	7.9 × 10e5 2.9 × 10e5 5.3 × 10e5 8.2 × 10e5 9.1 × 10e4		Lungs Heart Kidneys Liver Spleen	
2 Patient with systemic meningococcal disease (SMD) with shock	3.8 × 10^7^ copies/mL (plasma) 271 EU/mL (plasma) No spinal puncture was performed	10	FFPE	Lungs Heart Kidneys Liver Spleen	1.0 × 10e6 1.3 × 10e6 8.3 × 10e5 2.2 × 10e5 8.1 × 10e4		Lungs Heart Kidneys Liver Spleen	
3 Patient with systemic meningococcal disease (SMD) with shock	1.0 × 10^8^ copies/mL (serum) 2140 EU/mL (serum) Spinal puncture was performed post mortem. CSF contained 8 EU/mL	5 5	FFPE FF	Lungs Heart Kidneys Liver Spleen	2.1 × 10e7 4.6 × 10e7 3.2 × 10e6 5.8 × 10e5 1.1 × 10e5	2.4 × 10e8 4.2 × 10e6 6.3 × 10e7 ^*^ 5.9 × 10e6	Lungs Heart Kidneys Liver Spleen	Lungs Heart Kidneys Spleen
4 Patient with systemic meningococcal disease (SMD) with shock	3.0 × 10^7^ copies/mL (serum) 3800 EU/mL (serum) No spinal puncture was performed	2 2	FFPE FF	Lungs Heart Kidneys Liver Spleen	1.2 × 10e9 1.7 × 10e8 1.4 × 10e7 3.3 × 10e8 ^*^	2.3 × 10e8 6.1 × 10e7 8.3 × 10e7 1.2 × 10e8 4.3 × 10e7	Lungs Heart Kidneys Liver	Lungs Heart Kidneys Liver Spleen
5 Patient with systemic meningococcal disease (SMD) with shock	^*^ ^*^ ^*^	6 6	FFPE FF	Lungs Heart Kidneys Liver Spleen	1.5 × 10e7 ^*^ ^*^ ^*^ ^*^	4.1 × 10e7 3.5 × 10e7 9.9 × 10e6 1.7 × 10e7 ^*^	Lungs Heart	Lungs Heart Kidneys Liver Spleen
6 Control (Patients with non-infectious disease, sudden death)		15	FFPE		0 0 0		Lungs Heart Kidneys	
7^#^ Control (Patients with non-infectious disease, sudden death)		3	FFPE		0 0 0 0 0		Lungs Heart Kidneys Liver Spleen	

# *The control patient was an elderly smoker*.

* *not available*.

### Autopsy Procedure

The autopsy procedures were carried out at the Department of Pathology, Oslo University Hospital, Department of Pathology, Stavanger University Hospital, or at the section for Forensic Pediatric Pathology, Oslo University Hospital. Tissue samples from different organs were fixed in 4% buffered–neutral formalin at room temperature for 6–48 h, dehydrated, cleared, embedded in paraffin and cut in 4 μm thick sections. All tissue sections were routinely stained with hematoxylin and eosin (H&E). The FFPE sections were stored at room temperature.

### Subjects

#### Post Mortem Findings

Macro- and microscopic findings have previously been reported in patients No 1–5 (Brusletto et al., 2017). All five patients had hemorrhagic skin lesions and hemorrhagic adrenals.

#### Formalin-Fixed, Paraffin-Embedded (FFPE) Tissue From Patients With Meningococcal Shock and Multiple Organ Failure (Patients No 1–5)

The formalin-fixed, paraffin-embedded tissues were selected according to histopathological findings; presence of neutrophilic inflammatory infiltrates or thrombi. Small tissue specimens from five lungs, five hearts, four livers, four kidneys, and three spleens were available.

The samples were collected during the routine post mortem examination within 24 h after the patient died. The storage times of the FFPE tissue samples were 11, 10, 6, 5, and 2 years (Table 1).

#### Fresh Frozen (FF) Tissue From Patients With Meningococcal Shock and Multiple Organ Failure (Patients No 3–5)

Three lungs, three hearts, two livers, three kidneys and three spleens were collected in parallel with the routine post mortem examination and frozen at −80°C for later analysis. The storage times of the FF tissue were 6, 5, and 2 years. The samples had been partially thawed once and examined before this analysis (Hellerud et al., 2015).

#### Formalin-Fixed, Paraffin-Embedded (FFPE) Tissue From Patients With Acute Non-infectious Death (Controls) (Patients No 6 and 7)

FFPE tissues from two patients with a non-inflammatory disease were used as negative controls; patient No 6 died of a head injury and patient No 7 had a cerebral hemorrhage. The storage time of the specimens was 15 and 3 years, respectively. Tissue from lungs, heart, liver, spleen and kidneys were analyzed. No pathological findings were found in the tissues. The organ samples were collected at routine post mortem examination 24–48 h after death.

### Immunohistochemical Staining of FFPE Lungs and Heart Tissue Samples

IHC staining was performed on samples from patients 2, 3, and 7 (Table 1). Sections from lungs and heart were analyzed for presence of macrophages (CD68), neutrophils (MPO), T cells (CD3), and B cells (CD20). The inflammatory markers, plasminogen activator inhibitor-1 (PAI-1), presently also denoted serpine-1, and monocyte chemoattractant protein (MCP-1/CCL2), were also examined. The tissue samples were deparaffinized, rehydrated and demasked in a microwave oven for 24 min in Target Retrieval Solution or Tris/Edta buffer pH 9.1 for ab CD 68 and CD3. The primary antibodies used were anti CD3 1:50 (rabbit polyclonal ab 5690, Abcam), anti CD20 1:2500 (confirm anti-CD20 (L26) monoclonal mouse anti CD ready-to-use (RTU) cat. no 760-2531 Roche Diagnostics), anti CD68 1:3000 (monoclonal mouse anti human CD68 cat. no M0814 Agilent Technologies), anti MPO 1:1000 (rabbit polyclonal cat. no A0398, Agilent Technologies), anti PAI-1 1:400 (rabbit polyclonal ab 66705, Abcam) and anti MCP-1 1:400 (rabbit polyclonal ab9669, Abcam). Antigen-antibody reactions were visualized with DAKO EnVision horse radish peroxidase system (Agilent Technologies or DAKO Cytomation for MCP-1) using 3, 3′-diaminobenzidin as the chromogen. All tissue sections were counterstained with hematoxylin.

### Quantification of Immune Cells and Inflammatory Markers

Immunostained sections from heart and lungs from the patients and controls were examined using an Olympus BX51 microscope (obj. x20). Positively stained inflammatory cells were counted in 10 pictures (Diagnostic instruments. Inc, model 11.2 camera) from representative areas, a total area of 2 mm^2^ per section. The inflammatory markers (PAI-1 and MCP-1) were registered as present or not present.

### RNA Extraction

FFPE tissue samples for RNA extraction were cut using a microtome. A new sterile blade was used for each patient and washed with 70% ethanol between each organ block. The first sections of the tissue samples were discarded before cutting sections for RNA extraction. Freshly cut slices of two 10 μm-thick sections were isolated in parallel and immediately placed in 160 μL of deparaffinization Solution (cat. no: 19093 Qiagen) in a microcentrifuge tube. The miRNeasy FFPE kit (Qiagen, Hilden, Germany) was further used for extraction of total RNA in the QIAcube robot www.qiagen.com/MyQIAcube according to manufacturer's instructions. RNase–Free DNase I digestion step was added to remove DNA contamination and highly fragmented molecules in the RNA samples. The RNA was eluted in 20 μL RNase-free water. A negative control (sample without tissue sample) was subjected in parallel for isolation to check for contaminations. The total RNA samples were stored at −80°C before further analysis. All reactions were performed in an RNase-free environment; benches, instruments, and pipettes were cleaned and treated with RNaseZap solution (Ambion Inc., Austin TX) and RNase-free tips and microtubes were used. Total RNA isolated in parallel sections from FFPE tissue samples gave almost similar yields and purities (260/280 ratio) (data not shown). Therefore, a single tissue sample from each tissue was used for microarray analysis.

Approximately 30 mg of FF tissue samples were cut by a sterile scalpel and subjected for homogenization for 2 min in 700 μL QIAzol using a TissueLyzer (Qiagen, Hilden, Germany). Total RNA was furthermore extracted using the miRNeasy Mini Kit (Qiagen) and Phase Lock Gel™ Heavy (5 PRIME GmbH, Hamburg, Germany) and the QIAcube robot (www.qiagen.com/MyQIAcube) according to manufacturer's instructions. Total RNA was eluted in 50 μL RNase-free water and stored at −80°C before further analysis.

Total RNA concentration and purity (260/280 ratio) in each sample was determined with the NanoDrop ND-1000 Spectrophotometer (Thermo Fisher Scientific, Waltham, MA) and the quality assessed on Agilent 2100 Bioanalyzer RNA 6000 Nano Kits (Agilent Technologies, Palo Alto, CA).

### Microarray Analyses

Microarray analyses were performed using the Affymetrix GeneChip Human Transcriptome 2.0 Arrays (Affymetrix, Santa Clara, CA, USA). Total RNA (100 ng) was subjected to the Sensation Plus™ FFPE Amplification and WT Labeling Kit, following the manufacturer's protocol for whole–genome gene expression analysis. Biotinylated and fragmented single-stranded cDNAs were hybridized to the arrays. The arrays were washed and stained using an FS-450 fluidics station (Affymetrix, fluidics protocol FS450_0001). Signal intensities were detected by a Hewlett Packard (Palo Alto, CA, USA) 30007G gene array scanner. The scanned images were processed using the AGCC (Affymetrix GeneChip Command Console) software, and the CEL files were imported into Partek® Genomics Suite™ software (Partek, St.Louis, MO, USA) for statistical analysis. The Robust Multichip Analysis (RMA) algorithm was applied for generation of signal values and normalization. On each array 44,699 protein coding genes (transcript clusters) and 22,829 non–protein coding genes (including immature microRNAs) could be detected. Gene transcripts with maximal signal values of < 5 (log2) across all arrays were removed to filter for low and non-expressed genes. For expression comparisons of different groups, profiles were compared using a one-way ANOVA model. The results were expressed as fold changes (FC). Genes with FC ≥ |±2| and a *P*-value < 0.05 were regarded as significantly regulated. Partek Genomics Suite software was used to generate principal component analysis (PCA), Venn diagram and a table for top up- and down-regulated transcripts of the gene expression data. To minimize experimental artifacts RNA extraction, sample amplification and labeling, hybridization and washing, and scanning procedures, were carried out by the same operator.

### The Affymetrix Synthesis

Staggered concentrations of internal positive controls, poly A RNA controls, which monitor the entire labeling process, and hybridization controls, which monitor the hybridization process, were spiked into the Sensation Plus™ FFPE Amplification and WT Labeling Kit, and found as Present calls in all samples.

### Gene Expression Data Analysis

To summarize the information obtained in the microarray analysis and to check data quality, we used Principal Component Analysis (PCA) which identifies the directions (principal components) in which the variation in the data is maximal, and enables us to visualize this variation in a plot.

A Venn diagram illustrating the number of differentially expressed genes (one-way ANOVA, *p* < 0.05, FDR 5%) in FFPE tissue samples (lungs, heart, kidneys, liver and spleen) from meningococcal septic shock patients compared with controls, was performed using: http://bioinformatics.psb.ugent.be/webtools/Venn/.

The top 10 up- and down-regulated gene transcripts, differentially expressed in large organs from patients with meningococcal septic shock vs. controls were calculated and displayed in a table.

### Functional Analysis by Ingenuity Pathway Analysis (IPA)

Bioinformatics analysis was conducted on significantly regulated genes to identify biological functions/pathways that were most significantly associated with the data set by Ingenuity Pathways Analysis (IPA) (Ingenuity Systems, Redwood City, CA, USA). Briefly, the data set containing gene identifiers and corresponding fold changes and *p*-values was uploaded into the web-delivered application where each gene identifier was mapped to its corresponding gene object in the IPA Knowledge Base (IPKB). Fisher's exact test was performed to calculate *p*-values determining the probability that each biological function and/or disease assigned to the data set was due to chance alone. The program then computed a score for each network according to the fit of the network to the set of focus genes. The score was derived from a *p*-value which indicates the likelihood of the focus genes in a network being found together because of random chance. The Z-score indicates predicted activation state of the biofunctions, canonical pathways, and upstream regulators. The data sets were mined for significant pathways with the IPA library of canonical pathways and networks were generated by using IPA as graphical representations of the molecular relationships between genes and gene products.

Enrichment analysis was performed using IPA's ≪core analysis≫ for each tissue sample and a ≪comparison analysis≫ between the tissue samples. These functions have the ability to identify significantly activated biological functions and pathways, molecular functions, and relationships in our dataset of genes. A right-tailed Fisher's exact test calculated *p*-values corrected for multiple testing by the Benjamini-Hochberg method. In addition an “Upstream regulator” analysis was used to identify the cascade of upstream transcriptional regulators that were involved in MSS patients and whether they were likely activated or inhibited to obtain the observed gene expression profile changes in our datasets.

### Canonical Pathway Analysis in IPA

There are two main groups of canonical pathways in IPA: metabolic and signaling. These pathways are hierarchically grouped according to a number of sub-categories. In order to identify those pathways most relevant to the cell-types and disease of our study, disease- and cell-specific pathways not deemed relevant to our study were excluded from the analysis. Canonical pathways significantly enriched by the differentially expressed genes in the datasets were identified with the right-tailed Fisher's exact test, which calculates a *P*-value determining the probability that the canonical pathway is associated with the data set due to random chance alone. The *P*-values were corrected for multiple testing using the Benjamini-Hochberg method for correcting the false discovery rate (Benjamini and Hochberg, 1995).

### Validation of Genes by Real Time Quantitative Reverse Transcription PCR (qRT-PCR)

To validate the gene expression data from FFPE and FF tissue samples analyzed by microarray analysis, selected differentially expressed genes with the significantly highest fold change value were quantified by qRT-PCR (TaqMan gene expression assays and the Applied Biosystems ViiA7 sequence detection system). Total RNA (100 ng) was reverse transcribed using SuperScript™ VILO™ cDNA Syntheisis Kit (cat. no: 11754250) (ThermoFisher Scientific). In a total volume of 20 μL; 20 ng cDNA (2 μL), and 1 μL of either RT-PCR primer cat 4331182 (CCL2 Hs 00234140-m1, IL1R Hs 01073300-m1, CXCL8 Hs 00174103-m1, IL1B Hs 01555410-ml, IL6 Hs 00174131-mL, SERPINE 1 Hs 00167155-m1, MT1A Hs00831826-s1, RPL9 Hs 01552541-g1, PLA2G2A Hs 00179898-m1, HAMP Hs 00221783 –m1, DYRK2 Hs 00705109-s1) were added to 10 μL TaqMan Fast Advanced mastermix (Applied Biosystems, www.appliedbiosystems.com) and 7 μL of H_2_O. The relative changes of each transcript using the mean of DYRK2 (Hs 00705109-s1) as endogenous control, were calculated using the ViiA™7 Software v1.1 and the ΔΔCT method (Livak and Schmittgen, 2001).

### Validation of Proteins by Multiplex Assays

Homogenized FF tissue samples were used to examine whether mRNAs are translated into proteins. Fifty mg of tissue sample, 495 μL CytoBuster Protein Extraction Reagent (Novagen, San Diego, CA) and 5 μL Protease Inhibitor cocktail set I (Calbiochem, Darmstadt, Germany) was homogenized with Xiril Dispomix. After completion, the samples were incubated for 5 min on ice and thereafter centrifuged at 2500 x g for 20 min at 4°C. The supernatants were transferred to Nunc tubes (Thermo Fisher Scientific Inc., Waltham, MA) and stored at −80°C before analysis. The supernatants were analyzed using multiplex cytokine assay (Bio-Plex Pro™ Human Cytokine assay), (Bio-Rad Laboratories, Hercules, CA) by Luminex® Technology.

### Quantification of *N. meningitidis* DNA and LPS in Plasmas/Serum/CSF From Patients With Meningococcal Disease in Samples Collected on Hospital Admission (Table 1)

Heparin-blood was collected in LPS-free vacuum tubes, centrifuged, plasma pipetted off and aliquoted as described in detail earlier (Brandtzaeg et al., 1989a; van Deuren and Brandtzaeg, 2000). Quantification of *N. meningitidis* DNA was performed as previously described in detail (Ovstebo et al., 2004; Gopinathan et al., 2012). The detection limit was 10^3^ *N. meningitidis* DNA copies/mL.

Quantification of LPS in plasma/serum/ CSF was initially performed with an in house developed limulus amebocyte lysate (LAL) assay and later with Chromo-LAL (Associates of Cape Cod, USA) with a detection limit of 0.2 EU/mL. The serum level is on average 63% of the plasma level (Brandtzaeg et al., 1992; van Deuren and Brandtzaeg, 2000).

### Quantification of *N. meningitidis* DNA in FFPE and FF Tissue From Patients With Meningococcal Disease in Samples Collected Post Mortem (Table 1)

All FFPE tissue samples were prepared according to routine procedures at the Department of Pathology. FF tissue were collected in parallel with the routine post mortem examination and frozen at −80°C for later analysis. Quantification of *N. meningitidis* DNA was performed as previously described in detail (Brusletto et al., 2017).

### Statistical Analysis

GraphPad Prism, version 7 was used for Standard error of the mean (SEM), medians and correlation plots (GraphPad Software, Inc.). Fischer's exact test was used when appropriate.

## Results

### Evaluation of RNA and Affymetrix Synthesis

#### RNA Extraction: Yield and Purity

We compared the concentration, purity and RIN of RNA from the different tissues samples. The concentration of RNA isolated from FFPE tissue samples (2 × 10 μm) from five patients with meningococcal septic shock ranged from 59 to 624 ng/μL, (*n* = 21 median 119 ng/μL). The purity ranged from 1.62 to 2.00 (260/280 ratio) (*n* = 21 and median 260/280 ratio = 1.89). RIN ranged from 2.3 to 2.6 (median 2.5 in RIN).

The concentration of RNA isolated from FFPE tissue samples from controls (*non-infectious disease)* ranged from 41 to 439 ng/μL (*n* = 8 and median 62 ng/μL). The purity ranged from 1.66 to 1.91 (260/280 ratio) (*n* = 8 and median 260/280 ratio = 1.8. RIN ranged from 1.9 to 2.5 (median 2.4 in RIN). The concentration of RNA isolated from FF tissue samples (30 mg) from three patients with meningococcal disease ranged from 24 to 2762 ng/μL (*n* = 15 and median 716 ng/μL). The purity ranged from 1.96 to 2.08 (260/280 ratio) (*n* = 15 and median 260/280 ratio = 2.05). RIN ranged from 2 to 8.5 (median 3.5 in RIN).

Overall, these samples showed adequate RNA quantity and quality for further amplification and labeling using Affymetrix Sensation Plus FFPE kit allowing RIN values > 1.4 to be acceptable.

### Evaluation of the Gene Expression Profiles

#### Principal Component Analysis: Sources of Variability and Effect of Storage Time

Principal component analysis was used to determine the significant sources of variability, identify patterns in the data sets, and if storage time had effect on gene expression patterns. The PCA analysis (Additional File 1: Figure S1) indicated that the two groups, meningococcal septic shock patients and controls, cluster with minor overlap, indicating different gene expression patterns. The storage time of the tissue samples at the time of analysis varied greatly within the study population. The effect of degradation of the nucleic acids was evaluated using PCA. Despite low RIN values, the PCA plot (Additional File 1: Figure S1) indicates that the age of the tissue samples had no impact on the gene expression analysis, showing no clustering of young or old tissue samples.

### Gene Expression Data in FFPE Tissue Samples vs. FF Tissue Samples

**Figure 1:**
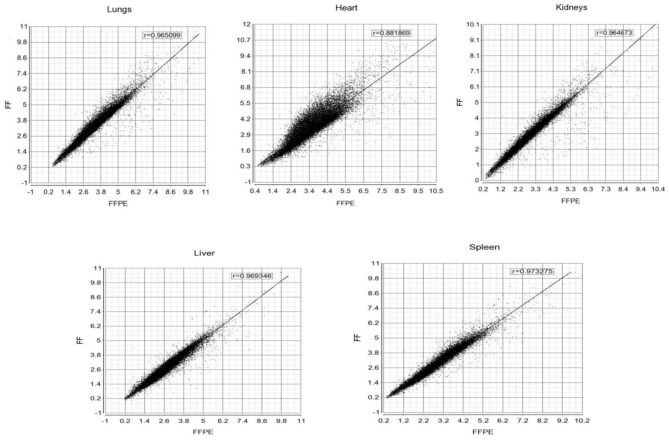
Correlation plots of the gene expression profiles between FF and FFPE tissue samples from meningococcal septic shock patients. The mean signal intensities for unfiltered gene expression (25000 transcripts) with NM (m-RNA) and NR (ncRNA) annotations on FF (x-axis) and FFPE (Y-axis) tissue samples for respectively lungs, heart, kidneys, liver and spleen. Pearson correlation coefficients (r) for each scatter plot are shown on the top.

The gene expression profiles from fresh frozen (FF *n* = 3) and formalin-fixed, paraffin embedded tissues (FFPE *n* = 5) tissue samples (lungs, heart, kidneys, liver, and spleen) from meningococcal shock patients were compared to identify if gene expression profiles were different between the storage methods. Correlation plots (Figure 1) of the gene expression profiles showed correlations ranging from *r* = 0.88 to 0.97, indicating good consistency between the methods.

The selected transcripts (CCL2, IL1R, CXCL8, SERPINE 1, MT1A, IL-6, RPL9, PLA2G2A, HAMP, IL-1B) quantified by qRT-PCR in FFPE and FF, showed correlation coefficients for lungs *r* = 0.896, heart *r* = 0.816, kidneys *r* = 0.936, liver *r* = 0.413 and spleen *r* = 0.521.

### Immunohistochemical Staining of Lungs and Heart

**Figure 2:**
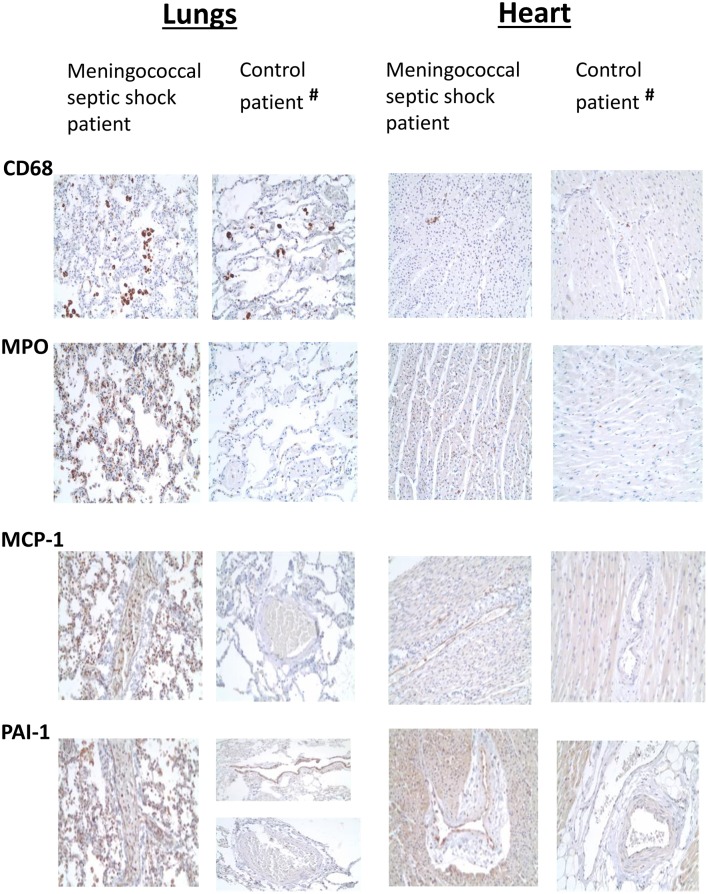
Immunohistochemical examination of FFPE tissue samples (lungs and heart) from one meningococcal septic shock patient and one control (acute non-infectious death). Immunohistochemical detection of CD68, MPO, MCP-1, and PAI-1 positive cells (labeled brown) in sections from lungs and heart from meningococcal septic shock patient and control patient with acute non-infectious death (Obj. x20) (^#^The control patient, elderly smoker).

Overall, the lungs had a higher number of infiltrative inflammatory immune cells than the heart. CD68-positive macrophages, MPO-positive neutrophils, CD3-positive T-lymphocytes and CD20–positive B-lymphocytes were present in higher numbers in the lungs and the heart of meningococcal septic shock patients as compared to the control (Figure 2 and Table 2). In the lungs, large CD68-positive macrophages were observed in the alveolar spaces whereas smaller CD68-positive cells were present within the alveolar walls and small vessels (Figure 2 and Table 2). More endothelial and inflammatory cells were significantly positive for PAI-1/SERPINEI and MCP-1 in the lungs and heart from the meningococcal septic shock patients compared with the controls (Figure 2).

**Table 2:** Immunohistochemically examination of FFPE tissue samples (lungs and heart) from two patients with meningococcal septic shock and one control patient (acute non-infectious death).

**IHC marker**	**Celltype**	**Meningoccal septic shock patient**	**Meningoccal septic shock patient**	**Meningoccal septic shock patient**	**Meningoccal septic shock patient**	**Control patient^**#**^**	**Control patient^**#**^**
		Lungs 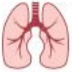	Heart 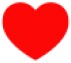	Lungs 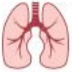	Heart 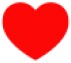	Lungs 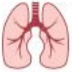	Heart 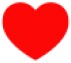
CD68	Macrophages/leukocytes	Large 160 Small 96	117	Large 189 Small 76	99	Large 57 Small 121	40
MPO	Neutrophil granulocytes	790	99	313	133	34	11
CD3	T-lymphocytes	290	37	117	29	139	8
CD20	B-lymphocytes	198	15	142	18	11	1

*Immunohistochemical quantification of positive cells of CD68, MPO, CD3 and CD20 in sections from lungs and heart from meningococcal septic shock patients and control patient with acute non-infectious death (^#^The control patient, elderly smoker). Quantification of average number of CD68, MPO, CD3, and CD20 positive cells per mm^2^*.

### Gene Expression Profiles in FFPE Tissue Samples From Meningococcal Septic Shock Patients and Controls

PCA was used to visualize variations in gene expression profiles from the different organs from meningococcal septic shock patients (*n* = 5) and controls (*n* = 2). Overall, the PCA plot demonstrated an organ specific gene clustering of the data sets (Additional File 2: Figure S2).

**Figure 3:**
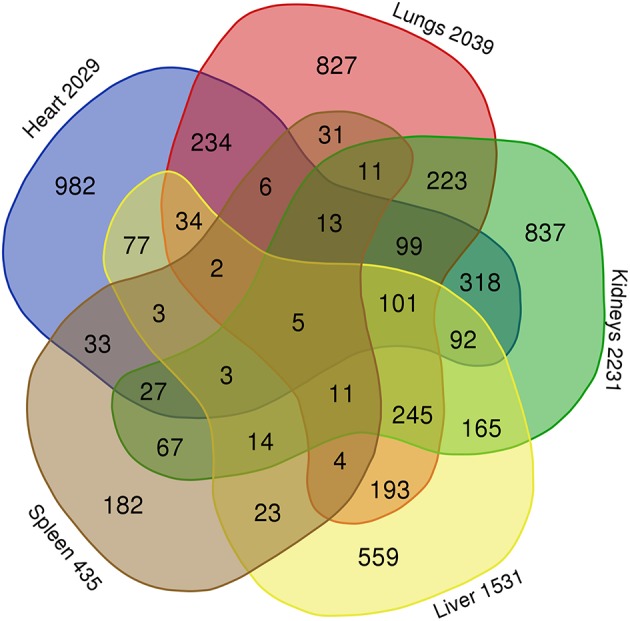
Comparison of differentially expressed gene transcripts in FFPE tissue samples (lungs, heart, kidneys, liver, and spleen) from meningococcal septic shock patients vs. control patients (acute non-infectious death). Gene transcripts with signal values of <5 (log2) across all arrays were removed to filter for low and non-expressed genes. For expression comparisons of different groups, the profiles were compared using a one way ANOVA model. *P*-value <0.05, correction FDR 5%. The Venn diagram illustrate that 827 gene transcripts were specific for the lungs of the 2039 differentially expressed gene transcripts, in heart 982 of 2029, in kidneys 837 of 2231, in liver 559 of 1531 and in spleen 182 of 435. Five gene transcripts were common for lungs, heart, kidneys, liver, and spleen.

A comparison of the lists from the transcripts from lungs, heart, kidneys, liver and spleen using a Venn diagram (Figure 3) showed 827 specific transcripts for lungs, 982 for heart, 837 for kidneys, 559 for liver and 182 for the spleen. Five transcripts including 1 mapped transcript; DMTF1, a transcription regulator in the senescent pathway and 4 transcripts with unmapped ID in IPA, were found to be common for these five organs.

The top 10 most up-and down-regulated differentially expressed transcripts in the organs (lungs, heart, kidneys, liver, and spleen) from patients with meningococcal septic shock vs. controls also showed an organ specific gene expression where transcripts for different classes of non-coding RNAs such as transfer RNA and piRNA were most pronounced in most organs (Table 3). In lungs and heart, also transcripts resulting in proteins involved in inflammation, were on the top 10 up-regulated list.

**Table 3:** Top 10 differentially expressed up-regulated (A) and down-regulated (B) gene transcripts in large organs from patients with meningococcal septic shock vs. controls.

**(A) Top 10 genes up-regulated**				
**Transcript ID**	**Gene symbol**	**mrna_assignment**	**(Nm-sepsis vs. Control)**	
			***p*-value**	**Fold change**
**LUNGS**				
TC16002035.hg.1	MT1A	NM_005946 // RefSeq //Homo sapiens metal lothionein 1A (MTlA), mRNA. // chr16 //100 //	0.004	13,526
TC06003084.hg.1	TNFAIP3	AY820830 // NONCODE // accn=AY820830 class=mRNAlike lncRNA name=NULL ref=H-invitational	0.029	10,872
TC17000383.hg.1	CCL2	NM_002982 // RefSeq // Homo sapiens chemokine (C-C motif) ligand 2 (CCL2), mRNA. // chr	0.008	10,100
TC17002194.hg.1	CSF3	NR_033662 // NONCODE // accn=NR_033662 class=lncRNA name=ref=RefGeneNoncode transcript	0.005	9,916
TCOY000203.hg.1		TCONS_00017657 // NONCODE // accn=NULL class lnciRNA=Human lincRNA ref=BodyMapLinc	0.007	9,546
TC07000643.hg.1	SERPINE1	NM_000602 // RefSeq // Homo sapiens serpin peptidase inhibitor, clade E (nexin, plasmin	0.009	7,339
TC16002075.hg.1	MT1L	NR_001447 // RefSeq // Homo sapiens metallothionein 1L (gene/pseudogene) (MT1L), non-co	0.007	7,125
TC0700190l.hg.1		DQ573766 // NONCODE // accn=DQ573766 class=piRNA name=piR-41878 ref=NONCODE v2.0 transc	0.011	7,122
TCOX001624.hg.1	SAT1	NR_027783 // NONCODE // accn=NR_027783 class=lncRNA name=ref=RefGeneNoncode transcript	0.017	7,106
TC16000469.hg.1	MT1JP	NR_035677 // RefSeq // Homo sapiens metallothionein 1J, pseudogene (MTIJP), non-coding	0.003	6,892
**HEART**				
TC01000807.hg.1		uc021ooz.1 // UCSC Genes // A nucleics Acid regulating cell growth. // chr1 //100 // 1	0.035	12,671
TC17000383.hg.1	CCL2	NM_002982 // RefSeq // Homo sapiens chemokine (C-C motif) ligand 2 (CCL2), mRNA.// chr	0.001	8,403
TC19000464.hg.1	HAMP	NM_021175 // RefSeq // Homo sapiens hepcidin antimicrobial peptide (HAMP},mRNA.// chr	0.004	8,385
TC01002205.hg.1	NPPB	NM_002521 // RefSeq // Homo sapiens natriuretic peptide B (NPPB), mRNA. // chr1 // 100	0.007	8,341
TC02003446.hg.1	IL1RL1	BC012580 // NONCODE // accn=BC012580 class=mRNAlike lncRNA name=NULL ref=H-invitational	0.001	8,306
TC04000408.hg.1	CXCL8	NM_000584 // RefSeq // Homo sapiens chemokine (C-X-C motif) ligand 8 (CXCL8), mRNA. //	0.000	8,184
TC0300060l.hg.1		uc021xcv.1 // UCSC Genes // Sequence 827 from Patent W02010139812. // chr3 // 100 // 10	0.024	8,121
TC07001900.hg.1		uc022aml.1 // UCSC Genes // Sequence 827 from Patent W02010139812. // chr7 // 100 // 10	0.024	8,121
TC14000567.hg.1		uc021ryn.1 // UCSC Genes // Sequence 827 from Patent W02010139812. // chr14 // 100 // 1	0.024	8,121
TC20000115.hg.1		uc021waz.1 // UCSC Genes // Sequence 827 from Patent W02010139812. // chr20 // 100 // 1	0.024	8,121
**KIDNEYS**				
TC07000285.hg.l		DQ597235 // NONCODE // accn=DQ597235 class=piRNA name=piR-35301 ref=NONCODE v2.0 transc	0.014	23,062
TC07001384.hg.1		DQ599872 // NONCODE // accn=DQ599872 class=piRNA name=piR-37938 ref=NONCODE v2.0 transc	0.014	23,062
TC07001404.hg.1		DQ599872 // NONCODE // accn=DQ599872 class=piRNA name=piR-37938 ref=NONCODE v2.0 transc	0.014	23,062
TC07001415.hg.1		DQ599872 // NONCODE // accn=DQ599872 class=piRNA name=piR-37938 ref=NONCODE v2.0 transc	0.016	21,738
TC01000006.hg.1		DQ597235 // NONCODE // accn=DQ597235 class=piRNA name=piR-35301 ref=NONCODE v2.0 transc	0.024	17,640
TC01001875.hg.l		DQ597235 // NONCODE // accn=DQ597235 class=piRNA name=piR-35301 ref=NONCODE v2.0 transc	0.024	17,640
TC01002068.hg.1		DQ599872 // NONCODE // accn=DQ599872 class=piRNA name=piR-37938 ref=NONCODE v2.0 transc	0.024	17,640
TC01002070.hg.1		DQ599872 // NONCODE // accn=DQ599872 class=piRNA name=piR-37938 ref=NONCODE v2.0 transc	0.024	17,640
TC01003864.hg.1		DQ599872 // NONCODE // accn=DQ599872 class=piRNA name=piR-37938 ref=NONCODE v2.0 transc	0.024	17,640
TC07000363.hg.1		DQ597235 // NONCODE // accn=DQ597235 class=piRNA name=piR-35301 ref=NONCODE v2.0 transc	0.024	17,640
**LIVER**				
TC15000159.hg.1		DQ575741 // NONCODE // accn=DQ575741 class=piRNA name=piR-43853 ref=NONCODE v2.0 transc	0.027	8,740
TC15000185.hg.1		DQ575741 // NONCODE // accn=DQ575741 class=piRNA name=piR-43853 ref=NONCODE v2.0 transc	0.027	8,740
TC1S000960.hg.1		DQ575741 // NONCODE // accn=DQ575741 class=piRNA name=piR-43853 ref=NONCODE v2.0 transc	0.027	8,740
TC15001139.hg.l		DQ575741 // NONCODE // accn=DQ575741 class=piRNA name=piR-43853 ref=NONCODE v2.0 transc	0.027	8,740
TC15001173.hg.1		DQ575741 // NONCODE // accn=DQ575741 class=piRNA name=piR-43853 ref=NONCODE v2.0 transc	0.027	8,740
TC09001260.hg.1		DQ592725 // NONCODE // accn=DQ592725 class=piRNA name=piR-59837 ref=NONCODE v2.0 transc	0.015	6,904
TC01002307.hg.1	PLA2G2A	NM 000300 // RefSeq // Homo sapiens phospholipase A2, group IlA (platelets, synovial fl	0.029	5,981
TC10000517.hg.1		DQ596518 // NONCODE // accn=DQ596518 class=piRNA name=piR-34584 ref=NONCODE v2.0 transc	0.004	5,830
TC07001415.hg.1		DQ599872 // NONCODE // accn=DQ599872 class=piRNA name=piR-37938 ref=NONCODE v2.0 transc	0.048	5,387
TC04000834.hg.1		uc021xue.l // UCSC Genes // A nucleics Acid regulating cell growth. // chr4 /1100 // 1	0.030	5,228
**SPLEEN**				
TC15000464.hg.1		uc021snh.1 // UCSC Genes // Annexin Il and uses thereof. // chr15 // 100 //100 // 0 //	0.049	10.048
TC1S000024.hg.1		DQ592939 // NONCODE // accn=DQ582939 class=piRNA name=piR-50051 ref=NONCODE v2.0 transc	0.025	7.334
TC1S000148.hg.1		DQ592939 // NONCODE // accn=DQ582939 class=piRNA name=piR-50051 ref=NONCODE v2.0 transc	0.025	7.334
TC15000207.hg.l		DQ592939 // NONCODE // accn=DQ582939 class=piRNA name=piR-50051 ref=NONCODE v2.0 transc	0.025	7.334
TC15000238.hg.1		DQ592939 // NONCODE // accn=DQ582939 class=piRNA name=piR-50051 ref=NONCODE v2.0 transc	0.025	7.334
TC15000243.hg.1		DQ592939 // NONCODE // accn=DQ582939 class=piRNA name=piR-50051 ref=NONCODE v2.0 transc	0.025	7.334
TC15001014.hg.1		DQ592939 // NONCODE // accn=DQ582939 class=piRNA name=piR-50051 ref=NONCODE v2.0 transc	0.025	7.334
TC15001053.hg.1		DQ592939 // NONCODE // accn=DQ582939 class=piRNA name=piR-50051 ref=NONCODE v2.0 transc	0.025	7.334
TC15001115.hg.1		DQ592939 // NONCODE // accn=DQ582939 class=piRNA name=piR-50051 ref=NONCODE v2.0 transc	0.025	7.334
TC15001129.hg.1		DQ592939 // NONCODE // accn=DQ582939 class=piRNA name=piR-50051 ref=NONCODE v2.0 transc	0.025	7.334
**(B) Top 10 genes down-regulated**				
**LUNGS**				
TC06000232.hg.1		uc021ypj.l // UCSC Genes // transfer RNA Val (anticodon AAC) // chr6 // 100 // 100 // 0	0.015	−11,569
TCOX000897.hg.1		uc022btp.l // UCSC Genes // transfer RNA Val (anticodon TAC) // chrX // 100 // 100 // 0	0.005	−10,920
TC11001821.hg.1		uc021qjo.l // UCSC Genes // transfer RNA Val (anticodon TAC) // chr11 // 100 // 100 //	0.005	−10,920
TC06000298.hg.1		uc021ytf.l // UCSC Genes // transfer RNA Arg (anticodon CCG) // chr6 // 100 // 100 // 0	0.021	−10,855
TC06001457.hg.1		uc021ysp.1 // UCSC Genes // transfer RNA Arg (anticodon CCG) // chr6 // 100// 100 // 0	0.021	−10,855
TC16000104.hg.1		ucQ21tbd.1 // UCSC Genes // transfer RNA Arg (anticodon CCG) // chr16 // 100 // 100 //	0.021	−10,855
TC17000843.hg.1		uc021ucu.l // UCSC Genes // transfer RNA Arg (anticodon TCG) // chr17 // 100//100 //	0.017	−10,120
TC06001414.hg.1		uc021yqx.l // UCSC Genes // transfer RNA Val (anticodon AAC) // chr6 // 100 // 100 // 0	0.006	−9,847
TC06000180.hg.1		uc021ymv.1 // UCSC Genes // transfer RNA Arg (anticodon TCG) // chr6 // 100 // 100 // 0	0.039	−9,792
TC06001445.hg.1		ucQ21yrz.1 // UCSC Genes // transfer RNA Arg (anticodon TCG) // chr6 // 100 // 100 // 0	0.038	−9,587
**HEART**				
TC02001958.hg.1		uc02lviy.l // UCSC Genes // Rfam model RF00005 hit found at contig region AC016700.8/16	0.045	−4,841
TC16000732.hg.1		uc021szx.1 // UCSC Genes // Rfam model RF00005 hit found at contig region AL022341.6/55	0.045	−4,841
TC11000663.hR.1		uc021qlw.1 // UCSC Genes // transfer RNA Ser (anticodon GCT) // chr11 // 100 // 100 //	0.026	−4,729
TC06001356.hg.1		uc021ymw.1 // UC5C Genes // transfer RNA Ser (anticodon GCT) // chr6 // 100// 100 // 0	0.043	−4,103
TC06001447.hg.1		uc021ysa.1 // UCSC Genes // transfer RNA Ser (anticodon GCT) // chr6 // 100 // 100 // 0	0.043	−4,049
TC15001239.hg.1		uc021sji.1 // UC5C Genes // transfer RNA Ser (anticodon GCT) // chr15 // 100 // 100 //	0.043	−4,049
TC06000239.hg.1		uc021yps.1 // UC5C Genes // transfer RNA Ser (anticodon GCT) // chr6 // 100 // 100 //0	0.043	−3,939
TC16000947.hg.1		uc021tes.1 // UCSC Genes // transfer RNA Leu (anticodon TAG) // chr16 // 100 // 100 //	0.037	−3,918
TC06000222.hg.1		ucQ21yow.1 // UC5C Genes // transfer RNA Ser (anticodon GCT) // chr6 // 100 // 100 // 0	0.044	−3,891
TC01000224.hg.1		ucQ21ohh.1 // UC5C Genes // transfer RNA Asn (anticodon GTI) // chr1 // 100 // 100 // 0	0.048	−3,830
**KIDNEYS**				
TC07000285.hg.1		DQ597235 // NONCODE // accn=DQ597235 class=piRNA name=piR-35301 ref=NONCODE v2.0 transc	0.012	−8,975
TC07001384.hg.1		DQ599872 // NONCODE // accn=DQ599872 class=piRNA name=piR-37938 ref=NONCODE v2.0 transc	0.012	−8,975
TC07001404.hg.1		DQ599872 // NONCODE // accn=DQ599872 class=piRNA name=piR-37938 ref=NONCODE v2.0 transc	0.003	−7,759
TC07001415.hR.1		DQ599872 // NONCODE // accn=DQ599872 class=piRNA name=piR-37938 ref=NONCODE v2.0 transc	0.014	−7,563
TC01000006.hg.1		DQ597235 // NONCODE // accn=DQ597235 class=piRNA name=piR-35301 ref=NONCODE v2.0 transc	0.003	−7,544
TC01001875.hg.1		DQ597235 // NONCODE // accn=DQ597235 class=piRNA name=piR-35301 ref=NONCODE v2.0 transc	0.003	−7,544
TC01002068.hg.1		DQ599872 // NONCODE // accn=DQ599872 class=piRNA name=piR-37938 ref=NONCODE v2.0 transc	0.003	−7,544
TC01002070.hg.1		DQ599872 // NONCODE // accn=DQ599872 class=piRNA name=piR-37938 ref=NONCODE v2.0 transc	0.049	−7,522
TC01003864.hg.1		DQ599872 // NONCODE // accn=DQ599872 class=piRNA name=piR-37938 ref=NONCODE v2.0 transc	0.006	−6,619
TC07000363.hg.1		DQ597235 // NONCODE // accn=DQ597235 class=piRNA name=piR-35301 ref=NONCODE v2.0 transc	0.006	−6,619
**LIVER**				
TC11001821.hg.1		ucQ21qjo.1 // UC5C Genes // transfer RNA Val (anticodon TAC) // chr11 // 100 // 100 //	0.026	−35,075
TCOX000897.hg.1		uc022btp.1 // UC5C Genes // transfer RNA Val (anticodon TAC) // chrX // 100 // 100 // 0	0.026	−35,075
TC06001414.hg.1		ucQ21yqx.1 // UC5C Genes // transfer RNA Val (anticodon AAC) // chr6 // 100 // 100 // 0	0.008	−32,753
TC06001418.hg.1		ucQ21yrg.1 // UC5C Genes // transfer RNA Val (anticodon AAC) // chr6 // 100 // 100 // 0	0.020	−32,128
TC05002164.hg.1		ucQ21yke.1 // UC5C Genes // transfer RNA Val (anticodon AAC) // chr5 // 100 // 100 // 0	0.020	−32,128
TC05001076.hg.1		uc021yjv.1 // UC5C Genes // transfer RNA Val (anticodon AAC) // chr5 // 100 // 100 // 0	0.020	−32,128
TC05001075.hg.1		uc021yjt.1 // UCSC Genes // transfer RNA Val (anticodon AAC) // chr5 // 100 // 100 // 0	0.020	−32,128
TC03000905.hg.1		uc021xhc.1 // UCSC Genes // transfer RNA Val (anticodon Me) // chr3 // 100 // 100 // 0	0.020	−32,128
TC06000194.hg.1		ucQ21ynm.1 // UC5C Genes // transfer RNA Val (anticodon CAC) // chr6 // 100 // 100 // 0	0.020	−31,406
TC05002166.hg.1		ucQ21ykg.1 // UC5C Genes // transfer RNA Val (anticodon CAC) // chr5 // 100// 100 // 0	0.020	−31,406
**SPLEEN**				
TC16000114.hg.1		uc021tbt.1 // UCSC Genes // transfer RNA Pro (anticodon AGG) // chr16 // 100 // 100 //	0.047	−10,448
TC11002109.hg.1		uc021qnm.1 // UCSC Genes // transfer RNA Pro (anticodon TGG) // chr11 // 100 // 100	0.035	−9,303
TC01001076.hg.1		ucQ21oty.1 // UC5C Genes // transfer RNA Asn (anticodon GTT) // chr1 // 100 // 100 // 0	0.024	−5,623
TC01003178.hg.1		ucQ21oxr.1 // UC5C Genes // transfer RNA Asn (anticodon GTT) // chr1 // 100 // 100 // 0	0.024	−5,623
TC06001058.hg.1		uc021zgl.l // UC5C Genes // transfer RNA Leu (antci odon TAA) // chr6 // 100 // 100 // 0	0.007	−4,800
TC01001870.hg.1		ENST00000365394 // ENSEMBL // RNA, 5S ribosomal pseudogene 77 [gene_biotype:rRNA transc	0.020	−4,307
TC03000086.hg.1		ENST00000362739 // ENSEMBL // RNA, 5S ribosomal pseudogene 124 [gene_biotype:rRNA trans	0.049	−4,244
TC16000173.hg.1		ucQ21tdf.1 // UC5C Genes // transfer RNA Thr (anticodon CGT) // chrl6 // 100 // 100 //	0.041	−4,242
TC15001029.hg.1		DQ583164 // NONCODE // accn=DQ583164 class=piRNA name=piR-50276 ref=NONCODE v2.0 transc	0.010	−3,899
TC18000391.hg.1		DQ583164 // NONCODE // accn=DQS83164 class=piRNA name=piR-50276 ref=NONCODE v2.0 transc	0.010	−3,899

*Gene transcripts with maximal signal values of < 5 (log2) across all arrays were removed to filter for low and non-expressed genes. The table displays transcripts ID (affymetrix ID), gene symbol; mrna assignment, P-values and Fold Changes values*.

### Descriptive Analysis and Predicted Biological Functions of Differentially Expressed Genes in FFPE Tissue Samples From Meningococcal Septic Shock Patients

Table 4A shows the numbers of differentially expressed genes in the meningococcal septic shock tissue samples compared to control tissue samples and their association with molecular, cellular, and physiological functions that were identified in lungs, heart, kidneys, liver, and spleen. Based on the number of differentially expressed genes, all tissue samples were affected, with least affection in the spleen. The highest number of affected molecules (Table 4B) was associated to cellular movement, cell to cell signaling and interaction and cell death and survival with lungs and heart as most influenced.

**Table 4:** Numbers of genes differentially expressed (A) and functional analysis (B) in organs from patients with meningococcal septic shock (*n* = 5) performed by IPA.

	**Lungs**	**Heart**	**Kidneys**	**Liver**	**Spleen**
**(A)**					
**Differentially expressed genes** *p* < 0.05	2039	2029	2231	1531	435
Up-regulated^a^	331	458	421	130	74
Down–regulated^a^	519	48	316	758	64
Mapped ID eligible for IPA-analysis^b^	850	506	737	888	138
**Analyse ready genes** Fold change >I2I, *p* < 0.05	171	223	164	207	39
**(B)**					
**Molecular and cellular functions**	Molecules^c^	Molecules^c^	Molecules^c^	Molecules^c^	Molecules^c^
Cellular movement	47	79		33	
Cell-To-Cell signaling and interaction	38	51			4
Cell death and survival	66	119	73	38	
Cellular development	56				
Cellular growth and proliferation	59				
Protein synthesis		44	37		
Gene expression		65	37		
Lipid metabolism				34	
Small molecular biochemistry				46	
Vitamin and mineral metabolism				19	
Cell morphology					
Cellular compromise			15		5
Molecular transport			24		
Amino acid metabolism					1
Cell cycle					5
Cellular assembly and organization					7
**Physiological system development and function**	Molecules^c^	Molecules^c^	Molecules^c^	Molecules^c^	Molecules^c^
Hematological system development and function	47	68	31		4
Immune cell trafficking	38	49	17		2
Tissue development	41	47			
Skeletal and muscular system development and function	25				
Cardiovascular system development and function	37		23	26	
Organismal survival		85	49		
Tissue morphology		64			
Organ morphology				14	3
Organismal development				40	7
Digestive system development and function				13	
Hepatic system development and function				10	
Embryonic development			16		
Reproductive system development and function					5

a *Functional analysis performed with IPA; filtering criteria: FC ≥|±2|, p < 0.05. P-value of overlap comparing values from meningococcal septic shock (n = 5) patients with controls (n = 2)*.

b *The genes eligible for IPA analysis are identified by Ingenuity Knowledge Base*.

c *Molecules may refer to any gene, RNA or protein*.

**Figure 4:**
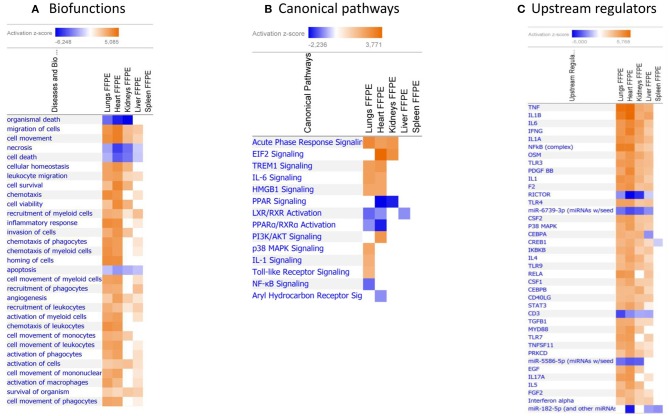
Predicted biofunctions **(A)**, canonical pathways **(B)**, and upstream regulators **(C)** in FFPE tissue samples from patients with meningococcal septic shock. ≪Comparison analysis≫ of biofunctions, canonical pathways, and upstream regulators significantly enriched in FFPE tissue samples from lungs, heart, kidneys, liver and spleen from meningococcal septic shock patients vs. controls patients (acute non-infectious death). The figure shows the most up-regulated biofunctions **(A)**, canonical pathways **(B)**, and upstream regulators **(C)** ranked according to expression levels in FFPE tissues from lungs. The Z-score indicates predicted activation state of the biofunctions, canonical pathways, and upstream regulators. Z-score value >|±1| are displayed. Blue color or lighter shades of blue indicate a negative Z-score and down-regulation of a biofunction, canonical pathways, and upstream regulators. Orange or lighter shades of orange indicates a positive Z-score and up-regulation of a biofunction, canonical pathways, and upstream regulators. Note that only the top pathways are shown.

**Figure 5:**
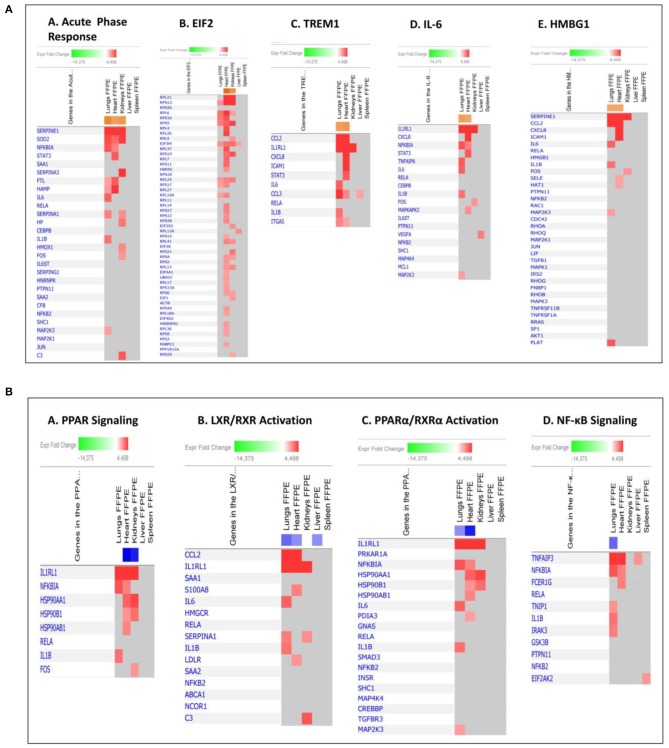
Predicted gene signaling pathways from the top up-regulated **(A)** and down-regulated **(B)** canonical pathways in FFPE tissue samples from patients with meningococcal septic shock vs. controls. The Z-score indicates predicted activation state of canonical pathways. Orange or lighter shades of orange indicate a positive Z-score and up-regulation of the pathway. Blue color or lighter shades of blue indicate a negative Z-score and down-regulation of the pathway. The transcripts in the gene signaling pathway are expressed as Fold Change (FC) values. Red or lighter shades of red indicates positive FC-values and up-regulation of transcripts, green color or lighter shades of green indicates negative FC- values and down-regulation of transcripts. Color gray indicates that a predicted activation state of a gene/transcript in the canonical pathway signaling network is not affected.

When gene expression changes in all the tissue samples from meningococcal septic shock patients were compared, the IPA “comparison analysis” predicted the down-regulated biofunctions to be organismal death, migration of cells, cell movement, necrosis and cell death (Figure 4A). The top regulated canonical pathways were predicted to be the acute phase response signaling, EIF2 signaling, TREM1 signaling, IL-6 signaling and HMGB1 signaling whereas PPAR signaling, LXR/RXR activation, PPARα/RXRα activation and NF-κB to be the most down-regulated (Figure 4B). Among predicted genes in the top five up-regulated canonical pathways, SERPINE 1 was on top in both acute phase signaling and HMGB1 signaling while MCP-1 was on top in TREM 1 and HMBG1 signaling (Figure 5A). Top predicted genes in the top down-regulated canonical pathways were IL1RL1, MCP-1, TNFAIP3, NFKB1A, and genes for the heat shock protein 90 family (Figure 5B). To identify the cascade of upstream transcriptional regulators that can explain the gene expression changes in our data sets, an upstream regulator ≪core analysis≫ was performed in IPA. The ≪core analysis≫, showed highly significant *p*-values (ranging from E35 to E3) (Table 5) of the top upstream regulators in the tissue samples. A ≪comparison analysis≫ of upstream regulators in the different tissue samples predicted TNF, IL1B, IL6, IFNG, and IL1A to be on top concerning up-regulation (Figure 4C) while RICTOR, CD3, and many miRNAs were down-regulated (Figure 4C).

**Table 5:** Top upstream regulators differentially expressed in organs from five patients with meningococcal septic shock^a^ vs. controls.

	**Lungs**	**Heart**	**Kidneys**	**Liver**	**Spleen**
Top upstream regulators *p*-value of overlap and predicted activation	TNF 2.32E-25 (Activated)	MYCN 3.39E-35 (Activated)	RICTOR 9.08E-17 (Inhibited)	RXRA 4.68E-11	CREB1 1.81E-04
	IL1B 7.77E-20 (Activated)	MYC 3.01E-24 (Activated)	NFE2L2 1.34E-09 (Activated)	ACOX1 7.64E-09	SBDS 3.45E-04
	IL-13 8.34E-20	IL1B 9.38E-21 (Activated)	MYCN 1.67E-09 (Activated)	PPARA 2.23E-07	miR-4668-3p 5.22E-04
	NFkB (complex) 4.81E-19 (Activated)	TNF 1.15E-20 (Activated)	IL1A 3.16E-08 (Activated)	APP 4.20E-07	miR-17-2-3p 5.91E-04
	IL1A 1.13E-18 (Activated)	IL3 2.47E-18 (Activated)	TNF 3.25E-08 (Activated)	PPARGC1A 4.32 E-07	ATF4 1.48-03

a *Functional analysis performed by IPA. P-values of overlap comparing values from meningococcal septic shock (n = 5) patients with controls (n = 2). Predicting activation or inhibition (filtering criteria: FC ≥|±2|, p < 0.05)*.

**Figure 6:**
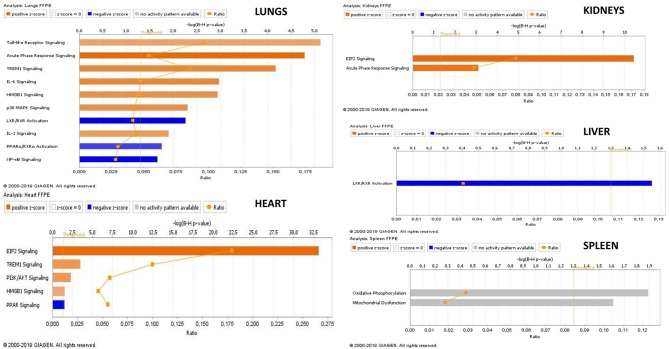
Transcriptional profiles of canonical pathways in FFPE tissue samples from meningococcal septic shock patients. The figure displays the top canonical pathways enriched in each organ. The mean values of *N. meningitidis* DNA/μg human DNA was in lungs: 2.5 × 10^8^, heart 5.4 × 10^7^, kidneys 4.6 × 10^6^ liver 8.3 × 10^7^, and spleen 9.4 × 10^4^. A ≪core analysis≫ was performed separately for each organ. Significantly enriched canonical pathways were identified with a right-tailed Fisher's Exact Test (*p* < 0.05, after correction for multiple testing using the Benjamini-Hochberg method). Ratio denotes the number of significantly expressed genes compared with the total number of genes associated with the canonical pathway. The Z-score=|±1| indicates predicted activation state of canonical pathway. Blue color or lighter shades of blue indicate a negative Z-score and down-regulation of the pathway, and orange or lighter shades of orange indicate a positive Z-score and up-regulation of the pathway. Gray color indicates no activity pattern available. Z-score value >|±1| are displayed.

**Figure 7:**
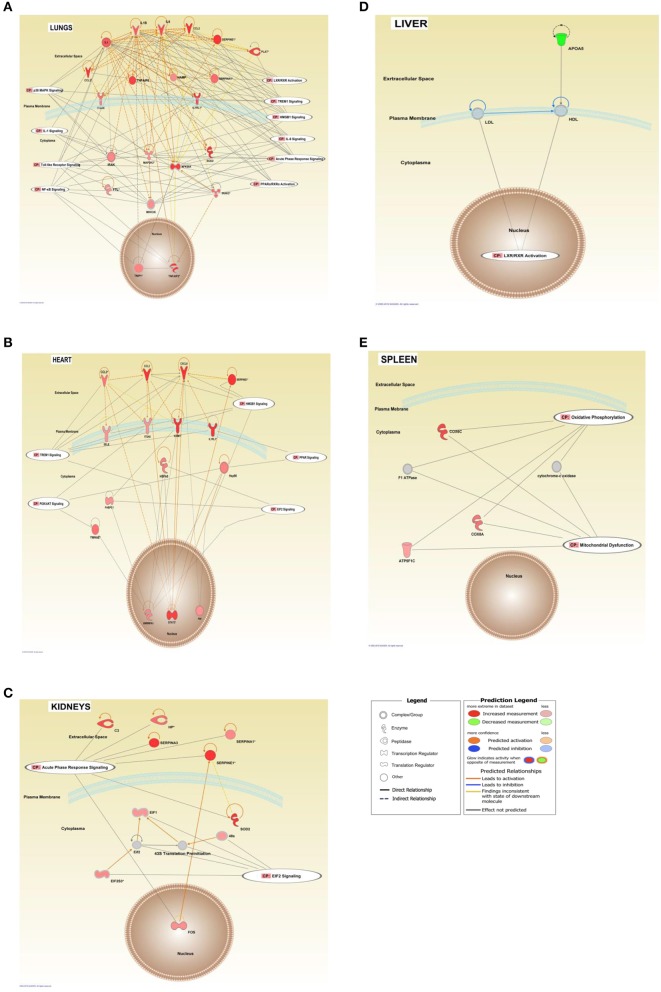
Interaction networks of top score canonical pathways and transcripts affected in FFPE tissue samples from meningococcal septic shock patients. The interaction networks in lungs **(A)**, heart **(B)**, kidneys **(C)**, liver **(D)**, and spleen **(E)** are generated through the use of IPA ≪core analysis≫. In addition, ≪Canonical Pathways Analysis≫ was performed to determine genes that were involved in well-documented canonical signal transduction or metabolic pathways, from the library of canonical pathways in IPA. Benjamini–Hochberg procedure for multiple testing corrections was performed. Significantly enriched genes in the dataset were overlayed into the specific canonical pathway. The analysis was performed *in silico* using Molecular Activity Predictor analysis of IPA. The shapes represent the molecular classes of the ≪gene≫. In the figures, red represents upregulation and green down-regulation, and color intensity represents the relative magnitude of change in gene expression. Gray color indicates no activity pattern available. Direct and indirect interactions are indicated by solid and dashed lines, respectively. The network diagram shows the biological relationship between the indicated genes lines: — represents direct physical interactions; —– represents indirect functional interactions; → represents activation; ⊣ represents inhibition. The blue lines indicate that the direction of regulation is consistent with IPA prediction. In contrast, yellow lines indicate that the regulation observed is inconsistent with expectations, while gray lines indicate lack of pre-existing data to formulate expectations. Nodes are displayed using various shapes that represent the functional class of the genes.

An IPA ≪core analysis≫ was performed separately for each organ to fine tune the top enriched pathways for each organs (Figures 6, 7A–E).

Our mRNA data in the lung tissue samples (Figures 6, 7A and Additional File 3: Table S1) showed a significant up-regulation of the Toll-like receptor-, acute phase response-, TREM1-, IL-6-, HMBG1-, p38MAPK-signaling, LXR/RXR activation, IL-1 signaling, PPARα/RXRα activation and NF-κB signaling pathways, resulting in striking up-regulations of several cytokines; CCL2, IL-6, IL1B, CCL3 (MIP-1α), transmembrane receptors; IL1RL, ITGA5, transcription regulators; NFKBIA, kinases; MAP2K3, IRAK3 peptidases; PLAT, enzymes; SOD2, FTL, TNFAIP3 and other molecules; such as IL1, SERPINE1, SERPINA1, HAMP, MKK3/6, TNFAIP6, and TNIP1.

The mRNA data in the heart tissue samples (Figures 6, 7B and Additional File 3: Table S2) showed a significant up-regulation of the EIF-2, TREM1-, P13/AKT-and HMGB1-signaling pathways and significant down-regulation of the PPAR signaling pathway (Figure 6) which led to activation of several cytokines; CCL2, CCL3, CXCL8, transmembrane proteins SELE (E-selectin), IL1RL1 (Interleukin 1 receptor –like 1) (ST2), ITGA5 (Integrin alpha-5), ICAM (Intracellular Adhesion Molecule), transcription regulator; STAT3, translation regulator; PABPC1, enzymes; HNRNPA1, HSPA5, and other molecules; Hsp90, IL1, SERPINE1, and YWHAE.

mRNA data in the kidneys tissue samples revealed a significant up-regulation of the EIF2 signaling pathways and the acute phase response (Figures 6, 7C and Additional File 3: Table S3) resulting in strikingly up-regulations of several peptidases such as; C3, HP, SOD2, transcription regulators; EIF1, EIF2, FOS, EIF2S3, and other molecules; such as SERPINE1, SERPINA1, SERPINA3 and 43S Translation Preinitiation.

### Validation of Gene Expression Profiles With qRT–PCR

We validated the microarray results with real time qRT-PCR, for genes that were significantly up-regulated in each tissue. Some of the genes were only up-regulated in one tissue, where as other genes were up-regulated in many tissues. The correlations were calculated from the fold changes in all the meningococcal shock patients compared with the controls, determined by qRT-PCR and microarray analysis giving the following results: for lungs *r* = 0.884 (MT1A, CCL2, SERPINE1), heart *r* = −0.997 (CCL2, HAMP, IL1RL1), kidneys *r* = −0.767 (CCL2, RPL9, CXCL8), liver *r* = 0.977 (PLA2G2A, SERPINE1, CXCL8) and spleen *r* = 0.376 (SERPINE1, MT1A, CCL2).

### Quantification of Selected Proteins in FF Tissue Samples From Meningococcal Septic Shock Patients

**Figure 8:**
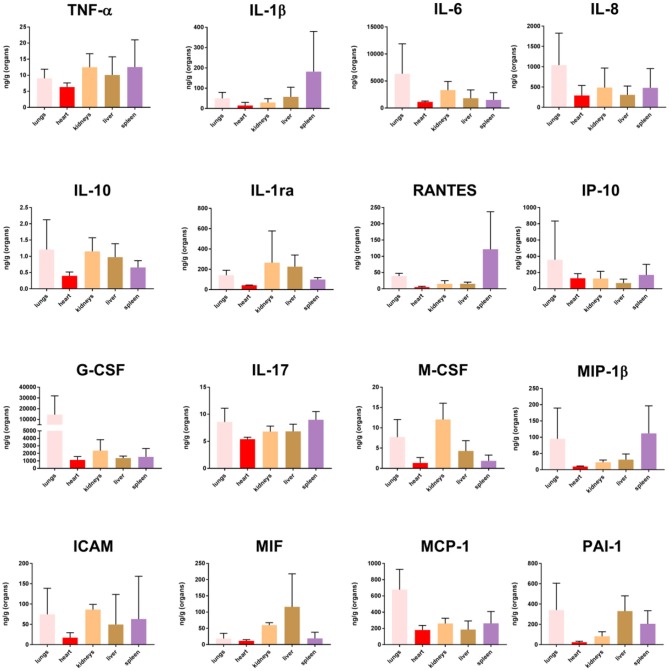
Cytokine concentration in fresh frozen tissue samples from patients with meningococcal septic shock (*n* = 3). The concentration unit is ng/g for the organ samples.

Proteins were selected for quantitation by Luminex multiplex analysis based high gene expression FC values when comparing tissues from MSS patients and controls (Figure 8). Tissue sample concentrations for 10 of the cytokines (TNF-α, IL-1β, Il-6, IL-8, IL-10, IP-10, G-CSF, IL-17, MIP-1β, and MCP-1) have previously been published in Hellerud et al. (2015) whereas IL-1ra, RANTES, G-CSF, IL-17, M-CSF, ICAM, MIF, and PAI-1 were analyzed for this publication. High levels of most cytokines, with individual variations, were found in the tissue samples (Hellerud et al., 2015).

### Quantification of *N. meningitidis* DNA and LPS in Plasma/Serum or CSF From Patients With Systemic Meningococcal Disease and Shock

The number of *N. meningitidis*/mL in the circulation of the patients with meningococcal shock ranged from 3.0 × 10^7^/mL to 2.8 × 10^8^/mL in plasma/serum (Table 1). LPS in plasma or serum ranged from 271 EU/mL to 3800 EU/mL (Table 1).

### Quantification of *N. meningitidis* DNA in FFPE and FF Tissue Samples From Patients With Systemic Meningococcal Disease and Shock

Copies of *N. meningitidis* DNA/μg human DNA in FFPE tissue samples in patients with systemic meningococcal disease and shock ranged from 8.1 × 10^4^ to 1.2 × 10^9^ (Table 1) and from 4.2 × 10^6^ to 2.4 × 10^8^ in FF tissue samples (Table 1) (Brusletto et al., 2017). The mean values of *N. meningitidis* DNA /μg human DNA was in lungs: 2.5 × 10^8^, heart 5.4 × 10^7^, kidneys 4.6 × 10^6^ liver 8.3 × 10^7^ and spleen 9.4 × 10^4^.

## Discussion

Post mortem tissue samples (FFPE) from patients with meningococcal septic shock provide an invaluable resource for profiling of gene expression and identifying affected molecular mechanisms. Our material is unique since both tissue samples frozen immediately after the autopsies as well paraffinized tissues stored at room temperature from the same organs in three of the five patients are included. This made it possible to study the influence of storage on the transcriptional profiles in samples stored from 2 to 6 years (Figure 1). The results suggest that FFPE tissues reflect fairly accurately the transcriptional profiles at the time of autopsy. Such studies have been lacking in gene expression studies of human bacterial infectious diseases. Specific proteins detected in the FFPE and FF tissue samples with immunohistochemistry techniques (Figure 2) or multiprotein assays (Figure 8), respectively manifested the regulation of the specific molecular pathways at protein levels.

Nucleic acids have historically been difficult to extract from FFPE tissue samples due to the need to remove paraffin and to counteract covalent protein-DNA interactions that results from the fixation process (Krafft et al., 1997; Farragher et al., 2008). The quality of nucleic acids from FFPE specimens may furthermore be affected by several other factors, such as variability in the time to tissue handling after onset of death (24–48 h), degree of autolysis (Gupta et al., 2012), archival storage time and various tissue sources (Chung et al., 2008; van Maldegem et al., 2008; Abdueva et al., 2010; Ferruelo et al., 2011). Isolating RNA has also been challenging since the FFPE archival methods may lead to chemical modifications and a partial degradation of the RNA (up to 50% of the RNA may not contain an intact poly-A-tail) (Farragher et al., 2008; Mittempergher et al., 2011) complicating established downstream analysis (Farragher et al., 2008). However, commercially available RNA extraction kits now enables to pick up fragmented RNA/cDNA isolated from FFPE tissue samples in a way that satisfy the quality and quantity needed for microarray analysis (Xiang et al., 2003; Zhu et al., 2016).

The quality control of isolated RNA (Boeckx et al., 2011; Ludyga et al., 2012; Seiler et al., 2016) from all the tissue samples in this study showed RNA integrity numbers (RIN) ≥1.9. The FFPE tissue samples ranged from 1.9 to 2–6 while the RIN of FF tissue samples ranged from 2 to 8.5. A RIN value of 1 indicates completely degraded RNA, whereas an RIN value of 10 indicates intact RNA. Our data indicate that the RNA isolated from the FFPE tissue samples from both meningococcal septic shock patients and controls, is heavily degraded. However, a RIN value of 1.4 and RNA purity measured as the 260/280 ratio of ≥1.8, has been considered as a minimum for successful completion of further microarray analyzes using the Affymetrix Sensation Plus FFPE amplification assay (Schroeder et al., 2006; Ribeiro-Silva et al., 2007; von Ahlfen et al., 2007).

Our study included FFPE tissue samples or fresh frozen tissue samples from five different organs from the same three meningococcal septic shock patients. To investigate the effect of the storage methods, the mean signaling intensity of the gene expression profiles from FFPE and FF tissue samples were compared and a correlation analysis resulted in r values ranging from 0.88 to 0.97 (Figure 1). These data indicate that RNA isolated from FFPE–and FF tissue samples give similar results and that microarray analysis enables to pick up and analyze the RNA molecules for gene expression profiles despite differences in storage time and low RIN values in the tissue samples. This is in line with other gene expression studies on FFPE stored cancer tissue samples (Linton et al., 2009; Zhu et al., 2016).

FFPE tissue samples from two persons with acute non–infectious death stored for 3 and 15 years were available as controls. A principal component analysis including information on storage times and storage methods (Additional File 1: Figure S1) indicated a clustering between the patient groups and that storage times and methods had minor effect on gene expression methodology, consistent with other studies (Thomas et al., 2013; Tyekucheva et al., 2015; Webster et al., 2015; Zhu et al., 2016). A PCA plot, a Venn diagram and a top 10 list of up-and down-regulated transcripts also suggests an organ specific transcriptional pattern clustering (Additional File 2: Figure S2, Figure 3 and Table 3). A ≪comparison analysis≫ in IPA was furthermore used to identify affected biofunctions and pathways in the different FFPE tissue samples and a disease associated specific tissue clustering was found in the septic-lungs, -hearts, -kidneys, -livers, and -spleens (Figures 4A–C) compared to controls. This in line with what is presented in Genotype-Tissue Expression (GTEx) - a database collecting gene expression data from numerous healthy and diseased tissue samples which observe tissue specific gene expression profiles (Carithers et al., 2015).

When comparing the multitude of gene expression results we see the contours of a very complex process resulting in a profound reprogramming with changes of gene expression in thousands of genes, both protein-coding and non-coding RNA transcripts. Many of these genes are related to the host's inflammatory response. However, also genes regulating metabolic processes, protein synthesis and folding, transmembrane calcium transport and mitochondrial functions, not directly related to inflammation, are altered probably as a consequence of inflammatory molecules with their multitude of effects. Notably, several non-coding RNAs, including long ncRNA, transfer RNA, piRNA and miRNA, were identified significantly regulated. These genetics elements are suggested to play essential roles in transcriptional and post-transcriptional regulation in innate immunity, mitochondrial- and organ dysfunction in trauma and septic patients (Ho et al., 2016a; Zhang et al., 2017). Given this complexity, it is impossible in this study, to pinpoint one single mechanism or genetic pathway which is the dominant cause of the organ dysfunction in the tissue samples. Our results suggest that involvement of many different genes and pathways may add up and the combined effect induce organ failure. The hypothesis that dysfunction of one “major” pathway leads to organ failure, may be too simplistic. We also find that the differential gene expression of these pathways differ from organ to organ (Figure 4). Some of our findings have previously been documented in a porcine meningococcal septic shock model (Hellerud et al., 2015), but not to our knowledge in human material (Ricote et al., 1998; Castrillo and Tontonoz, 2004; Pascual et al., 2005; Kidani and Bensinger, 2012; Soares et al., 2014; Khan et al., 2015; Hotchkiss et al., 2016; Balmer and Hess, 2017).

The main up-regulated bio-functions were involved in migration of cells, cell movement, cellular homeostasis, leukocyte migration, cell survival, chemotaxis, cell viability, and inflammatory responses while the main repressed cellular functions were associated with organismal death, necrosis, cell death and apoptosis (Table 4B, Figure 4A). Our data show that the pathophysiology observed in meningococcal septic shock tissue samples with different levels of *N. meningitidis* (Brusletto et al., 2017) may involve a complex interaction between the *N. meningitidis* and the host's immune system, most likely through recognition of LPS molecules embedded in the outer membrane of the bacteria. However, non-LPS bacterial molecules may also contribute to the inflammatory response (Hellerud et al., 2010).

The main up-regulated canonical pathways in our data set were acute phase response-, EIF-2-, TREM1-, IL-6- and HMBG1 signaling pathways (Figure 4B). Our study identified immunomodulating molecules such as SERPINE1 (PAI-1), CCL2 (MCP-1), CXCL8 (IL-8), ICAM, IL-6, SOD-2, NFKB1A, STAT3, IL1RL1, and RPL (Figure 5A) responding to the microbial invasion consistent with previous studies investigating dysregulated systemic inflammatory responses in sepsis/septic shock (Khan et al., 2015; Hotchkiss et al., 2016).

Among the most interesting findings was the down-regulation of pathways involving several lipid-activated transcription factors, namely nuclear receptors involved in activation of PPAR, LXR/RXR and PPARα/RXRα canonical pathways (Figures 4B, 5B). These are important for physiological lipid and cholesterol metabolism as well as for inflammation (Castrillo and Tontonoz, 2004). Short-term and mild changes in metabolism can positively modulate immune responses to eliminate pathogens and protect the host via disease tolerance (Soares et al., 2014; Van Wyngene et al., 2018). However, uncontrolled and severe disturbances of metabolic homeostasis are unfavorable (Balmer and Hess, 2017). The pathways are expressed in numerous tissues (Ricote et al., 1998) and have been shown to be implicated in the negative regulation of inflammatory responses to bacterial infections via anti-inflammatory or phagocytic roles by binding of transcription factors to co-repressor complexes and thereby maintaining inflammatory genes in a repressed state (Pascual et al., 2005; Kidani and Bensinger, 2012). Our results show that these pathways are variably down-regulated by *N. meningitidis* in the different organs (Figure 4B), indicating that the down-regulation might be a mechanism to dampen local inflammatory responses.

Quantification of cytokines in lysates, obtained from FF tissue samples from lungs, heart, kidneys, liver and spleen, showed that the organs under these circumstances might synthesize both pro- and anti -inflammatory cytokines in large quantities. Our data (Figure 8) demonstrated increased levels of selected cytokines in all tissue samples, with surprisingly high levels in the spleen. In the spleen, the transcriptional profiles changed minimally as compared with the transcriptional profiles in the other organs, given at the strictly chosen criteria in this study. This may possibly indicate a more profound posttranslatory regulation of the cytokine production in the spleen than in the other organs examined in this study. Most organs probably release these inflammatory mediators into the circulation. High levels of TNF, IL-1, IL-6, IL-10, and chemokines have been measured in plasma from patients with meningococcal sepsis (Waage et al., 1989b; Brandtzaeg et al., 1996b; Hazelzet et al., 1996; Moller et al., 2005; Brandtzaeg, 2006). A notable observation in this study is that comparatively much lower quantities of IL-10, the major inhibitor of proinflammatory cytokines are detected in different tissues as compared with blood levels (Lehmann et al., 1995; Brandtzaeg et al., 1996b; Hellerud et al., 2015). This may imply that interactions between pro- and anti-inflammatory molecules operating at tissue levels are quantitatively different in organ tissues as compared with the circulation. Importantly, many cytokine receptors are released in plasma during meningococcal septic shock which presumably dampen the proinflammatory immune reaction (van Deuren et al., 1995, 1997). However, we have no knowledge about the release of these receptors in the different tissues and the functional inhibitory effects of such release.

### Transcriptional Profiles in Lung Tissue Samples

Pulmonary function is heavily affected during meningococcal disease with capillary leakage leading to increased intra-alveolar fluid, pulmonary edema, tachypnea, and respiratory failure (Ferguson and Chapman, 1948; Pathan et al., 2003). The lung capillary cells are “bombarded” by a range of proinflammatory mediators including cytokines and chemokines possibly partly produced locally or conveyed via plasma. Our results suggest that the lungs appear to be key organs for intravascular leukocyte adherence during meningococcal sepsis. This is in line with the results from a porcine model (Hellerud et al., 2015). A striking observation was that the neutrophils in the meningococcal septic shock patients were detected within the alveolar walls and small vessels, as compared to patients with bacterial pneumonias where the neutrophils are located in the alveolar spaces. Leukopenia, particularly a low neutrophils count, is a striking laboratory observation in the meningococcal septic shock patients (Hazelzet et al., 1996; Brandtzaeg et al., 2001). Immunohistochemical analysis of lung tissues in this study documents the accumulation on neutrophils, T-lymphocytes and macrophages in the lung capillaries (Table 2 and Figure 2). A previous study of meningococcal shock patients suggests that the neutrophils are activated simultaneously with an intravascular adherence reflected by high plasma levels of neutrophil-specific elastase (Brandtzaeg and Kierulf, 1992). SERPINA1 (alpha-1-antitrypsin), a serine protease inhibitor which is up-regulated in this study, may dampen the proteolytic effect of the neutrophil-specific elastase (Janciauskiene et al., 2018). However, in the complex interplay between proinflammatory and prothrombotic factors, the capillary integrity appears to be negatively influenced resulting in an increased transcapillary flux of filtrated plasma, accumulating in the alveoli and causing pulmonary edema. PAI-1 (SERPINE 1) transcripts, involved in fibrinolysis, were markedly up-regulated as a part of the inflammatory response (Figure 7A). This inhibition of the fibrinolysis was counter balanced by other molecules such as PLAT (tPA) (Figure 7A and Additional File 3: Table S1). However, the balance between formation of thrombi vs. fibrinolysis appears to be tilted toward thrombosis in capillaries in different organs which was observed in the lungs of patient 2 (Brusletto et al., 2017). Our results are in line with an endotoxemic animal study (Semeraro and Colucci, 2000-2013) showing increased levels of PAI-1 mRNA in multiple organs.

CCL2 (MCP-1) detected in the septic lung tissue samples (Figure 7A and Additional File 3: Table S1), is a key chemoattractant protein that regulates migration and infiltration of monocytes/macrophages, and is synthesized after activation of the TREM1, HMBG1 signaling, and LXR/RXR activation pathways. The transmembrane receptors IL1RL1 and ITGA5, previously found to be regulating chemokines as CCL2 and CCL3, are also implicated in the pathogenesis of lung injury (Oshikawa et al., 2002; Akhabir and Sandford, 2010; Sarangi et al., 2012; Hellerud et al., 2015; Hotchkiss et al., 2016). Several inhibitors/modulators of the proinflammatory response such as NFKB1A-, TNFAIP3-, TNFAIP6-, TNIP1-, and IRAK3 mRNA (Figure 7A and Additional File 3: Table S1) were found to be regulated in lungs of the studied patients, suggesting modulation of the macrophages in the lungs from pro- to an anti-inflammatory phenotype. These molecules are associated with poor outcome (Pino-Yanes et al., 2011; Mittal et al., 2016; Jimenez-Sousa et al., 2019).

The MAP2K3 gene in the p38 MAPK signaling pathway, was significantly up-regulated in the lung tissue samples (Figures 6, 7A). This pathway has widespread effects in the pathophysiology of multiorgan dysfunction in septic shock in lungs (Asaduzzaman et al., 2008) as well as myocardium in meningococcal septic shock (Pathan et al., 2011). Autophagy by binding of LPS to TLR4 and inducing MAPK/p38 signaling, is found to be a pathway which is protective against multiple organ injuries in a murine sepsis models by preventing apoptosis, maintaining a balance between the productions of pro- and anti-inflammatory cytokines, and preserving mitochondrial functions (Ho et al., 2016b).

Iron is an essential factor required for the *N. meningitidis* bacteria to colonize and cause disease in humans (Ali et al., 2017). Iron hemostasis in the lungs was affected via up-regulation of FTL in our study (Figure 7A and Additional File 3: Table S1), an iron storage protein as well as an important regulator which diminishes inflammation and increases the anti-inflammatory response (Fan et al., 2014). Furthermore, the up-regulated genes SOD2 and HAMP, also have roles against oxidative stress and inflammatory cytokines from incoming neutrophils, as well as to preventing iron from being requisitioned by invading bacteria (Schmidt, 2015).

The extent of infiltration of inflammatory cells varies among tissue samples, but overall we found the greatest influx of cells in the lungs (Figure 2 and Table 2). This is consistent with our gene expression results that showed extensively pro-inflammatory responses in the lung tissue samples. The results also support our previous data which demonstrated a higher number of *N. meningitidis* in lung tissue samples compared with the other tissues (Brusletto et al., 2017). Other studies based on FFPE tissue samples have confirmed attachment of meningococci to the endothelial cells in different FFPE tissue organs using immunohistochemistry and PCR assays (Guarner et al., 2004; Mairey et al., 2006). The adherence of monocytes, which have turned into inflammatory macrophages, and presence of neutrophils, are facilitated by up-regulation of various adhesion molecules (ICAM, VCAM, E-selectin) on the endothelial cells and circulating leukocytes (E-selectin) (Hellerud et al., 2015). We found that PAI-1 and MCP-1, were located to the epithelium (Figure 2). Our results are consistent with an *E.coli* induced sepsis model in baboons which detected neutrophil granulocytes and macrophages on the endothelial cells in the lungs (Tang et al., 2007). Concomitantly PAI-1 was up-regulated in lungs (Silasi-Mansat et al., 2010). CCR2, the receptor for MCP-1 (CCL2) has also been found up-regulated in macrophages in lungs from patients with sepsis-induced lung injury (An et al., 2009).

### Transcriptional Profiles in Heart Tissue Samples

In industrialized countries heart failure combined with persistent hypotension are the primary causes of death in meningococcal disease. Acute heart failure has previously been attributed to inflammatory foci in the myocardium (Ferguson and Chapman, 1948; Dacosta et al., 1991; Neveling and Kaschula, 1993; Garcia et al., 1999). Troponin I, a specific marker of myocardial impairment and cell death, increased within 48 h of hospital admission of meningococcal shock patients and was related to a clinical severity score (Thiru et al., 2000). During the last 40 years, the circulatory collapse in fulminant meningococcal septicemia has been regarded as a consequence of declining vascular resistance and hypovolemia combined with an acute inflammation-induced cardiac failure (van Deuren et al., 2000; Stephens et al., 2007; Brandtzaeg and Van Deuren, 2012).

Myocardial depression is a well-recognized manifestation of organ dysfunction in sepsis. Excessive formation of nitric oxide (NO), reactive oxygen species (ROS) or nitrogen radicals, and transcriptional and metabolic changes have been proposed to explain the dysfunction (Martin et al., 2019) by either affecting a transient rise in cytosolic calcium (Ca^2+^) or a decrease in the cardiac contractile forces that defines septic cardiomyopathy (Martin et al., 2019). Endoplasmic reticulum (ER) stress due to activation of the unfolded protein response (UPR) via EIF-2 signaling pathway may also lead to myocardial apoptosis contributing to cardiac contractile dysfunction (Ceylan-Isik et al., 2010). The EIF signaling pathway, extensively affected in the septic heart tissue in our study (Figures 6, 7B) is known to moderate the synthesis of multiple molecules that are produced during a bacterial infection and to dampen ER stress (Nakayama et al., 2010; Shrestha et al., 2012). Several long non-coding RNAs (lncRNAs) (data not shown), were also significantly regulated in the septic heart samples. Such lncRNAs are in a rat model, suggested play an interaction and regulation role in the pathogenesis of sepsis-induced myocardial depression (Zhang T. N. et al., 2019).

The enzymes HSPA5 (BiP), HNRNPA1 and the transcription regulator PABPC1 previously found to be significantly expressed in response to ER stress (Dudek et al., 2009) and during gram-negative sepsis, may all induce autophagy (Xu et al., 2007; Ho et al., 2016b) and were substantially up-regulated in our meningococcal shock tissue samples (Figure 7B and Additional File 3: Table S2).

Both the TREM-1 and the HMBG1signaling pathways were up-regulated in the heart (Figures 6, 7B). They converge to amplify an inflammatory response by increasing the synthesis of pro-inflammatory mediators such as MCP-1, IL-8, IL1R1, and ICAM1. These molecules may also lead to binding of leukocytes to endothelial surfaces and disruption of the structures that maintain the integrity of the endothelium in a tissue. The transmembrane receptors SELE (E-selectin), ITGA5 and IL1R1 (ST2), all known (Hotchkiss et al., 2016; Hakanpaa et al., 2018; Szekely and Arbel, 2018) to recruit and enable leukocytes to roll along surfaces, were significantly up-regulated in our data set (Figure 7B and Additional File 3: Table S2). Studies of patients with meningococcal septic shock, has previously shown elevated levels of ICAM1 in the blood (Baines et al., 1999) while increased levels of IL1R1 correlates with poor prognosis in cardiovascular failure (Sabatine et al., 2008). Increased presence of macrophages and neutrophils in the heart tissue samples expressing IL-8, MCP-1 and PAI-1 (SERPINE1 gene) was confirmed by quantification of IL8 by Luminex and MCP-1 and PAI-1 by immunohistochemically methods (Figures 2, 8 and Table 2). *In vitro* studies of LPS-treated myocardium suggest that ICAM1 and VCAM1 contribute to myocardial dysfunction independent of neutrophil accumulation (Raeburn et al., 2002).

A dysregulation of the fibrinolytic system in the heart tissue samples due to significant up-regulation of SERPINE1 (PAI-1) was observed (Figures 2, 7B, 8 and Additional File 3: Table S2). No fibrin clots were observed histochemically. However, the presence of PAI-1 was found in cells in the heart tissue samples, suggesting that a reduced removal of fibrin may occur, leading to a deposition of fibrin clots in small blood vessels and inadequate tissue perfusion and organ failure (Brandtzaeg et al., 1990; Madoiwa et al., 2006). Previous studies indicate that elevated PAI-1 in plasma or serum is a significant predictor of disease severity and mortality, first discovered in plasma samples from meningococcal sepsis patients (Brandtzaeg et al., 1990; Kornelisse et al., 1996; Tipoe et al., 2018).

Damage-associated molecular pattern (DAMP) proteins such as High Mobility Group Box 1 (HMGB1), S100 proteins (S100A8/A9) and IL-1α, shown in our data to be significantly regulated, may all contribute to a host's defense by interacting through pattern recognition receptors (PRRs) such as RAGE and TLR4. HMGB1 has a pleiotropic role in inflammation and may be either secreted by activated macrophages and monocytes at a late pro-inflammatory stage, or released through cell necrosis and apoptosis (Wang et al., 1999; Bertheloot and Latz, 2017). HMBG1 has also an important role in the pathogenesis of cardiac dysfunction through increased ROS levels (Zhang et al., 2014).

The PI3K/Akt pathway (Figures 6, 7B), significantly up-regulated in the heart tissue samples, may negatively regulate NF-kB and probably limit pro-inflammatory and apoptotic events in monocytes/macrophages to protect the myocardium (Pourrajab et al., 2015). In a study with endotoxemic mice the P13K/AKT signaling pathway suppressed LPS–induced inflammation and coagulation (Schabbauer et al., 2004). In addition, YWHAE (14-3-3ε) was found up-regulated (Figure 7B and Additional File 3: Table S2). This is a protein that tightly regulates cellular and tissue homeostasis (Kosaka et al., 2012), and has been shown to have a role in inhibiting apoptosis in cardiomyocytes (Xing et al., 2000).

The LXR/RXR signaling pathway was significantly down-regulated in the heart tissue samples (Figures 4B, 5B). It involves the gene S100A8 that acts together with S100A9 to form the calprotectin protein, with both pro–and anti-inflammatory properties (Wang et al., 2018), as well as it exerts antimicrobial function by the ability to bind and control Zn^2++^ and Mn^2++^ required for bacterial growth (Damo et al., 2013). Calprotectin has a role in cardiovascular disease, and acts as an amplifier of the inflammatory response (Schiopu and Cotoi, 2013). The median calprotectin level in plasma from 13 patients in a meningococcal septic shock study was 20-fold higher than controls (Johne et al., 1997).

The PPAR signaling pathway was also significantly down-regulated in heart tissue samples. The nuclear receptors, particularly PPARs regulate cardiac fatty acid oxidation (FAO). In sepsis-mediated cardiac dysfunction a reduced energy production due in part to compromised FAO (Drosatos et al., 2011, 2013) and glucose catabolism (Tessier et al., 2003) will occur. In response to LPS and bacterial infections, down-regulation of PPARs and reduced FAO levels have been found in heart, liver and kidneys (Beigneux et al., 2000; Feingold et al., 2008; Drosatos et al., 2013). In children with septic shock decreased PPARα expression in whole blood correlates with severity (Standage et al., 2012).

### Transcriptional Profiles in Kidney Tissue Samples

Severe renal failure sets in gradually during bacterial sepsis and significantly contributes to the mortality of late-phase sepsis (White et al., 2013; Hotchkiss et al., 2016). Immunohistochemical examination of postmortem kidney tissues from patients with severe sepsis, reveals heterogeneous and rather nonspecific findings (Takasu et al., 2013). The cellular and molecular pathways are not well-understood (Hato et al., 2019).

In meningococcal septic shock, activation of the coagulation system and inhibition of the fibrinolytic system are so pronounced that thrombosis of the glomerular capillaries appears to be the principal cause of the acute renal failure. Patient 1, 2 and 4 (kidney samples from patient 5 was not microscopically examined) had thrombotic lesions of the glomeruli leading to acute reduced glomerular filtration and necrosis of the proximal tubuli (Brusletto et al., 2017). Several serine proteases were significantly up-regulated in the kidneys (Figures 6, 7C and Additional File 3: Table S3). Most strikingly were SERPINE1 (PAI-1) inhibiting fibrionolysis (Malgorzewicz et al., 2013), and SERPINA3 (α1-antichymotrypsin) and SERPINA1 (α1-antitrypsin), both known to affect the renin-angiotensin system in various ways (Schmaier, 2002; Zhu et al., 2007).

Our results may differ from septic shock studies caused by other pathogens with less pronounced coagulopathy (Brandtzaeg, 2006). The microarray results are in line with other studies in which the kidneys are exposed to endoplasmic reticulum (ER) stress and unfolded protein response (UPR) during severe pathological and inflammatory conditions (Khan et al., 2015). In a gram-negative animal sepsis model, the EIF1/EIF2a transcription regulators were mediators of translation initiation block in late-phase sepsis when transcriptomic changes were examined at multiple time points (Hato et al., 2019). Recent research indicates that ER stress is a major factor in renal tubular cell apoptosis resulting from ischemic acute kidney injury (Xu et al., 2016). The hypotension combined with reduced cardiac output will have a dramatic negative effect on renal function because of inadequate perfusion (Dickhout et al., 2011).

Our data also show activation of the complement system via C3, a component at the convergence of all three activation pathways in the complement system, leading to lysis and opsonophagocytosis when C3 is cleaved to C3b. The complement system is massively up-regulated in blood in meningococcal septic shock patients and directly associated with a lethal outcome (Brandtzaeg et al., 1989b, 1996a).

The SOD2 transcript was up-regulated in the kidneys. SOD2 is one of the oxidative stress genes that may counteract the effects of incoming neutrophils in the tissue (Macdonald et al., 2003). Up-regulation of this gene was observed in the lung tissue in a *E.coli* sepsis baboon model (Zhu et al., 2007). Haptoglobin (HP) is another oxidative stress gene found up-regulated. This high-affinity hemoglobin-binding protein and antioxidant may prevent degradative enzymes from gaining access to hemoglobin and thereby prevent loss of iron through the kidneys (Zager et al., 2012).

### Transcriptional Profiles in Liver Tissue Samples

The liver has critical roles during infections associated with removal of intravascular bacteria and LPS, i.e., bacteria scavenging, detoxifications by acyloxyacyl hydrolase (AOAH) and alkaline phosphatase and synthesizing acute-phase proteins (Poelstra et al., 1997; Shao et al., 2007; Deng et al., 2013). The response to the PAMPS is a highly regulated process involving several cells present in the liver such as hepatocytes, Kupffer cells, sinusoidal endothelial cells, primarily under the influence of cytokines such as TNF, IL-6 and IL-1β, (Nesseler et al., 2012). A combination of the cytokines are widely involved in acute phase protein production in the liver (Dhainaut et al., 2001).

The mRNA data in the liver tissue samples (Figures 6, 7D and Additional File 3: Table S4), showed a significant down-regulation of the LXR/RXR activation signaling pathway involving HDL, ApoA5 and LDL. These genes, involved in lipid uptake and cholesterol efflux, may be controlled by peroxisomes proliferation-activated receptors (PPARs) and liver X receptors (LXRs) (Zelcer and Tontonoz, 2006). Top upstream regulators in the liver tissue samples (Figure 4C) were TNF, IL1B and IL6. mRNA of AOAH and alkaline phosphatase were not significantly altered.

### Transcriptional Profiles in Spleen Tissue Samples

The genes of the spleen involved in septic shock (Figures 6, 7E and Additional File 3: Table S5) were to our surprise altered less than the genes in the other organs examined. The numbers of *N.meningitidis* in spleen tissue were also lower than in the other septic tissue samples (Table 1). Oxidative phosphorylation and mitochondrial dysfunction were affected and this may be explained by multiple organ failure following sepsis, possibly representing an adaptive state during which the organs “shut down” their normal metabolic functions to protect themselves from the overwhelming and prolonged insult. The decrease in energy supply due to mitochondrial inhibition may also trigger a “hibernation like-state” (Levy, 2007; Azevedo, 2010). Our top-ten list from the spleen tissue samples also showed an up-regulation of several piRNAs, molecules found to be involved in gene silencing and regulatory mechanisms (Parhad and Theurkauf, 2019; Zhang X. et al., 2019). Is the low number of genes regulated an indication of a dysfunctional spleen? Clinical experience suggests that these patients are not more inclined to contract other types of life threatening invasive bacterial infections before or after contracting *N.meningitidis* leading to meningococcal septic shock. Could the low number of regulated genes in the spleen represent an evolutionary adaptation mechanism since a key function of the spleen is removal of live and disintegrated bacteria from the circulation?

### Upstream Regulators

**Figure 9:**
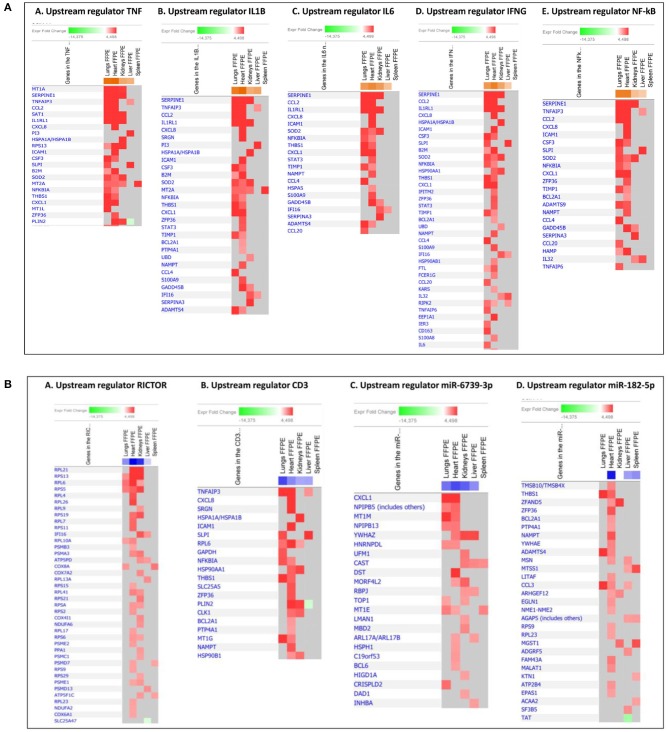
Predicted gene signaling network from the top up-regulated **(A)** and down-regulated **(B)** upstream regulators in FFPE tissue samples from patients with meningococcal septic shock vs. controls. The Z-score indicates predicted activation state of genes in the upstream regulator signaling network. Orange or lighter shades of orange indicate a positive Z-score and up-regulation of the upstream regulator. Blue color or lighter shades of blue indicate a negative Z-score and down-regulation of the upstream regulator. The transcripts in the signaling network are expressed as Fold Change (FC) values. Red or lighter shades of red indicates positive FC-values and up-regulation of transcripts, green color or lighter shades of green indicates negative FC-values and down-regulation of transcripts. Color gray indicates that a predicted activation state of a gene/transcript in the upstream regulator signaling network is not affected.

Prediction of top upstream transcription regulators in our tissue samples were found to be TNF, IL1B, IL6, IFNG, NFκB (complex), RICTOR, several miRNAs and CD3 (Figure 4C and Table 5), regulating signaling pathways in a sequential way. TNF, IL1B, IL6, IFNG, and NFκB (complex) are predicted to regulate cell survival signaling pathways, proliferation and metabolic processes. In addition, TNF can trigger programmed cell death (Varfolomeev and Vucic, 2018) (Figures 4C, 9A). The production of these upstream regulators is consistent with upstream regulators found in lung tissue samples in an *E.coli* sepsis baboon model (Zhu et al., 2007).

RICTOR, involved in regulation of cell growth and survival, may be inhibited due to overproduction of proinflammatory cytokines and cell survival (Cheng et al., 2016) (Figures 4C, 9B). Regulation of microRNA has been shown in several sepsis studies, reducing inflammatory cytokines and inhibition of cellular apoptosis (Ho et al., 2016a). Our data show several immature microRNAs to be down-regulated in our tissue samples. miR-6739-3p the most regulated in our data set, involved e.g., up-regulation of the chemokine CXCL1 (GRO-α) (Figures 4C, 9B), by recruiting and activating neutrophils for microbial killing at the tissue site (Sawant et al., 2016). miR-182-5p was found to be associated with down-regulation of multiple proteins in several tissue samples, however, most extensively in heart (Figures 4C, 9B). Regulation of miR-182-5p has been observed both in metabolic pathways, in cardiomyocytes and in sepsis (Vasilescu et al., 2009; Zhang et al., 2016, 2018).

Down-regulation of the upstream regulator CD3 was most strikingly observed in the lung tissue samples. CD3, associated with T-cell receptors that activate T-lymphocytes, has also been reported to be down-regulated in blood samples from other septic shock studies (Venet et al., 2005; Cazalis et al., 2014). However slightly higher numbers of CD3 positive immunostained cells were detected in both lungs and heart from meningococcal septic shock patients as compared to controls (Table 2).

## Conclusions

Our study demonstrates that organismal death and multiple defense mechanisms, both pro-and anti-inflammatory, were heavily activated in tissue samples from our patients. Genes associated with several non-inflammatory pathways related to basic metabolism and energy production showed reduced expression. These observations possibly represent a complex preprogrammed response to the massive amounts of meningococci in order to balance the homeostasis in the inflamed organs.

Septic lungs showed the most extensive gene expression changes whereas tissues from the spleen revealed, unexpectedly, fewer regulated genes. The different organs showed a quite specific transcriptional signature. These signatures reflect the activation of thousands of specific genes in resident cells and of immune cells attracted to the different organs from the circulation. Collectively they represent the cellular mRNA “fingerprint” associated with a lethal outcome in meningococcal septic shock and multiple organ failure induced by the massive proliferation of *N. meningitidis*. The results suggest that the acute organ failure is a combined effect of multiple genes being up- or down-regulated in concert and not changes in one single pathway. Future studies will add details that may explain more clearly the contribution of protein-coding and non-coding RNA transcripts in the pathophysiology of meningococcal septic shock and possibly identify new intervention methods.

## Data Availability Statement

The datasets supporting the conclusions of this article are available in the Gene Expression Omnibus (GEO) repository https://www.ncbi.nlm.nih.gov/geo/ under the identifier GSE141864 in accordance with minimum information about a microarray experiment (MIAME) standards.

## Ethics Statement

The study was approved by the Regional Medical Ethical Committee of South East Norway (2011/1413C Translational research, meningococcal disease and 2011/753 Studies of invasive meningococcal and pneumococcal disease). The patients' samples were collected after informed consent from patient parents or relatives and according to the Helsinki declaration. The Director of Public Prosecutions approved the use of forensic material for this research.

## Author Contributions

BB, RØ, PB, BH, JB, UG, OO, EL, and IG conceptualized and designed the study. EL and BH contributed with patient data and paraffin blocks from their hospital. BB, IG, EL, and RØ performed the laboratory experiments. BB, RØ, and PB performed the statistical analysis and drafted the manuscript. BB and OO performed the data analysis. BB, RØ, PB, JB, BH, UG, EL, and IG critically revised the manuscript. RØ, PB, and JB supervised the study. All authors read and approved the final manuscript.

### Conflict of Interest

The authors declare that the research was conducted in the absence of any commercial or financial relationships that could be construed as a potential conflict of interest.

## Abbreviations

ApoA5: Apolipoprotein A5
C3: Complement component 3
CD: cluster of differentiation
CREB: cAMP response element-binding protein
CSF: Cerebrospinal fluid
CXCL2: Chemokine (C-X-C motif) ligand 2 (alias MIP-2α)
CXCL8: chemokine (C-X-C motif) ligand 8 (alias IL-8)
CXCL10: (C-X-C) motif chemokine 10 (alias IP-10)
DAMPs: Damage-associated molecular patterns (alias alarmin)
DIC: disseminated intravascular coagulation
DYRK2: Dual specificity tyrosine-phosphorylation-regulated kinase 2
EIF2: Eukaryotic Initiation Factor 2
ER: endoplasmic reticulum
EU: Endotoxin unit
FF: fresh frozen
FFPE, formalin-fixed: paraffin-embedded
FOS, Fos proto-oncogene: AP-1 transcription factor subunit
FTL: Ferritin light chain
G-CSF: Granulocyte-colony stimulating factor (alias CFR 3)
HAMP: Hepcidin
HDL: High-density lipoprotein
HMBG1: High mobility group box 1 protein
HNRNPA1: Heterogeneous nuclear ribonucleoprotein A1
HP: Haptoglobin
Hsp90: heat shock protein 90
HSP90AA1: Heat Shock Protein 90 Alpha Family Class A Member 1
HSP90AB1: Heat Shock Protein 90 Alpha Family Class B Member 1
HSP90B1: Heat shock protein 90kDa beta member 1
HSPA5: heat shock 70 kDa protein 5 (alias BiP)
ICAM: intercellular adhesion molecules (alias CD 54)
IFNG: Interferon gamma
IHC: Immunohistochemistry
IL: interleukin
IL1RL1: Interleukin 1 receptor-like 1 (ST2)
IL1RN: interleukin-1 receptor antagonist (IL-1RA)
IP-10: Interferon gamma-induced protein 10
IPA: Ingenuity Pathway Analysis
IRAK-3: Interleukin-1 receptor-associated kinase 3
ITGA5: Integrin alpha-5
ITGB1: Integrin beta-1
LAL: limulus amebocyte lysate
LDL: Low-density lipoprotein
lncRNA: long non-coding RNA
LPS: lipopolysaccharides
LXR: liver X receptor
MAP2K3: Dual specificity mitogen-activated protein kinase kinase 3
MCP-1: Monocyte chemoattractant protein-1 (alias CCL2)
M-CSF: macrophage colony-stimulating factor (alias CSF1)
MIF: Macrophage migration inhibitory factor
MIP: Macrophage Inflammatory Proteins
miR: microRNA
miRNA: microRNA
MOF: multiple organ failure
MPO: Myeloperoxidase
MSS: meningococcal septic shock
mTOR: mammalian target of rapamycin
MYCN: N-myc proto-oncogene protein
MyD88: Myeloid differentiation primary response 88
NFKB1A: NF-Kappa-B Inhibitor Alpha
NF-κB: nuclear factor kappa-light-chain-enhancer of activated B cells
NmDNA: *N. meningitidis* DNA
NO: nitric oxide
P13/AKT: phosphoinositide 3-kinase (PI3K)/AKT
PABPC1: Polyadenylate-binding protein 1
PAI-1: Plasminogen activator inhibitor-1 (alias SEPINE 1)
PAMP: Pathogen-associated molecular pattern
PCA: Principal Component Analysis
piRNA: piwi-interacting RNA
PLAT: Tissue plasminogen activator (alias tPA)
PPAR: peroxisome proliferator-activated receptors
PPARA: Peroxisome proliferator-activated receptor alpha
PPARGC1A: Peroxisome proliferator-activated receptor gamma coactivator 1-alpha
RAGE: receptor for advanced glycation endproducts
RANTES, regulated on activation: normal T cell expressed and secreted (alias CCL5)
RICTOR: Rapamycin-insensitive companion of mammalian target of rapamycin
RIN: RNA integrity number
ROS: reactive oxygen species
RPL: L ribosomal proteins
RXR: retinoid X receptor
RXRA: Retinoid X receptor alpha
S100A8: calcium-binding protein A8 (alias Calgranulin A)
S100A9: calcium-binding protein A9
SELE, E-selectin, SERPINA1: Serpin Family A Member 1 (alias Alpha-1-antitrypsin)
SERPINA3: Serpin Family A Member 3 (alias Alpha 1-antichymotrypsin)
SMD: systemic meningococcal disease
SOD-2, Superoxide dismutase 2: mitochondrial
STAT3: Signal transducer and activator of transcription 3
TLR4: Toll-like receptor 4
TLRs: Toll-like receptor family
TNF: tumor necrosis factor
TNFAIP3, Tumor necrosis factor: alpha-induced protein 3
TNFAIP6: Tumor Necrosis Factor-Inducible Gene 6 Protein (alias TSG-6)
TNIP1: TNFAIP3-interacting protein 1
TREM1: Triggering receptor expressed on myeloid cells 1
UPR: unfolded protein response
VCAM-1: vascular cell adhesion molecule 1
YWHAE: Tyrosine 3-Monooxygenase/Tryptophan 5-Monooxygenase Activation Protein Epsilon (alias 14-3-3 protein epsilon).
