# Supplementary material for: Extensive Changes in Transcriptomic “Fingerprints” and Immunological Cells in the Large Organs of Patients Dying of Acute Septic Shock and Multiple Organ Failure Caused by Neisseria meningitidis
Source: Front Cell Infect Microbiol. 2020 Feb 19;10:42. doi: 10.3389/fcimb.2020.00042 (PMC7045056; doi:10.3389/fcimb.2020.00042)
Supplement: Supplementary file 1 [file Data_Sheet_1.pdf]

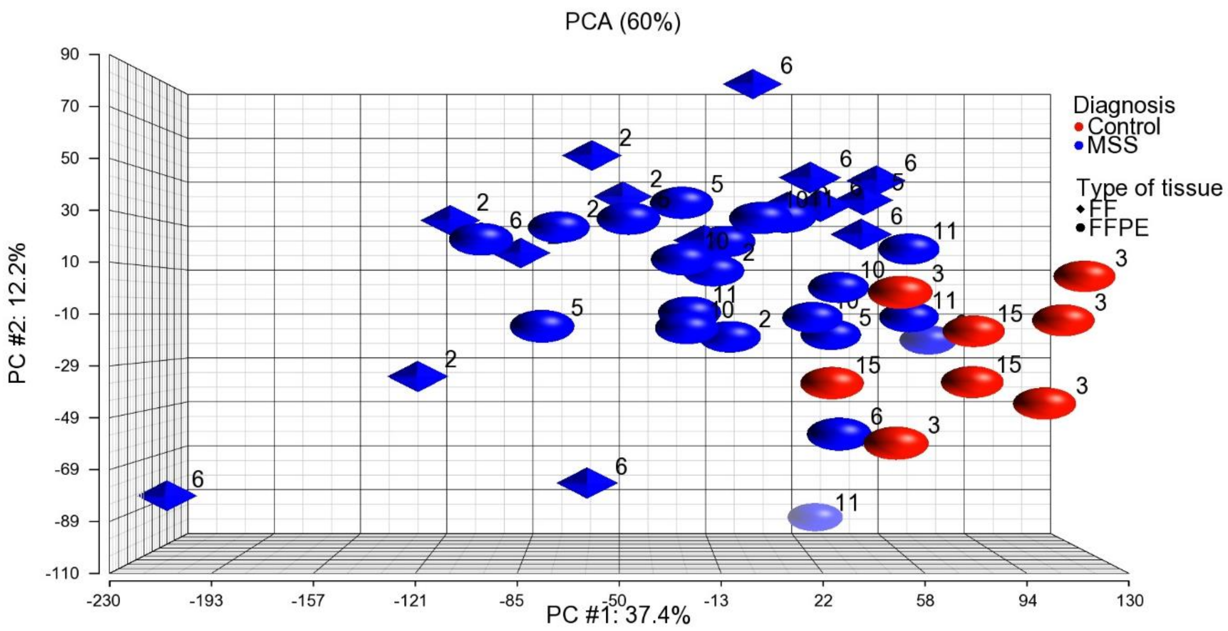

Additional file 1: Figure S1.

Effect of storage methods and time of FFPE and FF tissue samples

PCA of the gene expression profiles induced in organs from meningococcal septic shock and control patients (acute non-infectious death). Gene transcripts with maximal signal values of < 5 (log2) across all arrays were removed to filter for low and non-expressed genes. FFPE tissue samples are labelled with circles and FF tissue samples are labeled with pyramids.

Blue circles/pyramids represent gene expression in individual tissue samples from MSS patients. Red circles represent control tissue samples. The distance between circles/pyramids represents differences in the gene expression patterns. The numbers represents storage time (years) of tissue sample at RNA isolation time point. The percentage value describes the proportion of the total variance described by each principle component (PC) axis (PC1, PC2 and PC3). The three PCs explain 60 % of the variance in the data.

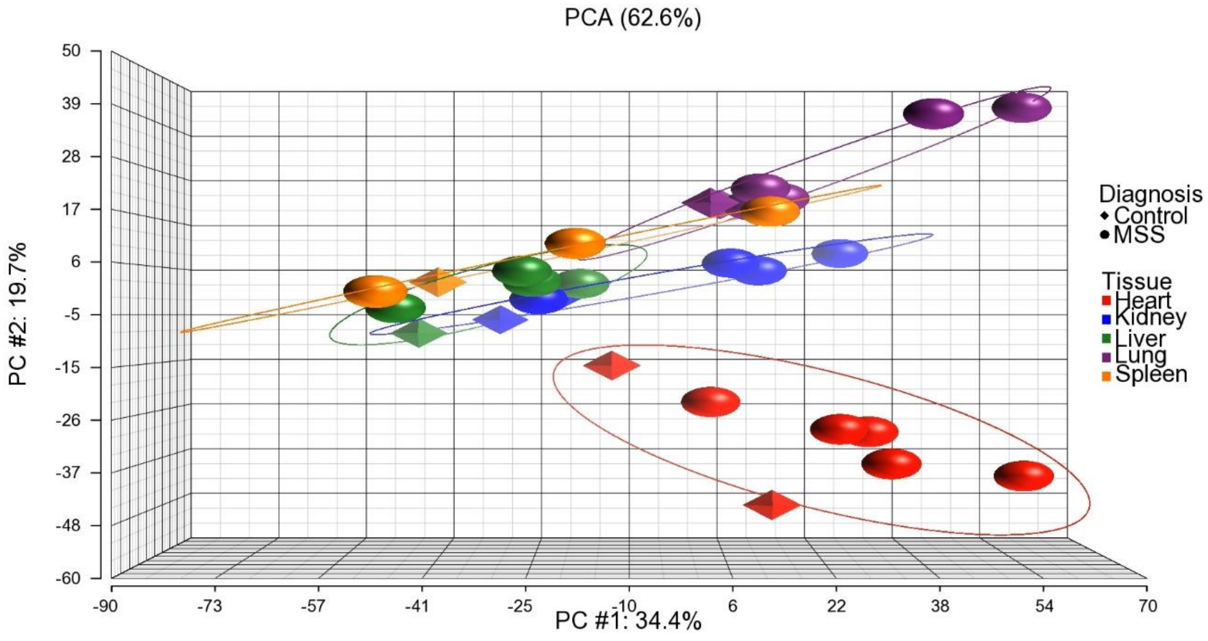

Additional file 2: Figure S2.

Gene expression in FFPE tissue samples from different organs.

PCA of the gene expression profiles induced in five different types of FFPE tissue samples from meningococcal septic shock patients (n=5) (circle) and controls (acute non-infectious death, n=2) (pyramid). Gene transcripts with maximal signal values of < 5 (log2) across all arrays were removed to filter for low and non-expressed genes. For expression comparisons of different groups, profiles were compared using a one way ANOVA model. p-value 0.05, correcting for false positive transcript a FDR of 5% was set.

Each circle and pyramid represents individual samples (red=heart, blue=kidney, green=liver, purple=lung, yellow=spleen), and the distance between them represent differences in the gene expression patterns. The percentage value describes the proportion of the total variance described by each principle component (PC) axis (PC1, PC2 and PC3). The three PCs explain 62,6 % of the variance in the data.

Symbol	p-value	Fold Change	Location	Family
**TNFAIP3**	0.029	10.9	Nucleus	enzyme
**CCL2**	0.008	10.1	Extracellular Space	cytokine
**SERPINE1**	0.009	7.3	Extracellular Space	other
**CCL3**	0.030	4.8	Extracellular Space	cytokine
**IL1RL1**	0.040	4.7	Plasma Membrane	transmembrane receptor
**SOD2**	0.025	4.0	Cytoplasm	enzyme
**NFKBIA**	0.041	3.8	Cytoplasm	transcription regulator
**PLAT**	0.038	3.7	Extracellular Space	peptidase
**TNFAIP6**	0.035	3.7	Extracellular Space	other
**IL6**	0.029	3.5	Extracellular Space	cytokine
**IL1B**	0.028	3.1	Extracellular Space	cytokine
**IRAK3**	0.042	2.9	Cytoplasm	kinase
**SERPINA1**	0.030	2.8	Extracellular Space	other
**ITGA5**	0.039	2.7	Plasma Membrane	transmembrane receptor
**TNIP1**	0.017	2.4	Nucleus	other
**HAMP**	0.010	2.2	Extracellular Space	other
**MAP2K3**	0.009	2.2	Cytoplasm	kinase
**FTL**	0.043	2.1	Cytoplasm	enzyme
**IL1**			Extracellular Space	group
**IRAK**			Cytoplasm	group
**MKK3/6**			Cytoplasm	group

Additional file 3: Table S1.

Transcripts affected in FFPE lung tissue samples from meningococcal septic shock patients

Data obtained from a “core analysis “ in IPA (Fig. 7A), (FC ≥ |±2|, p<0.05, BH 1.3 and Z-score |±1|).

Symbol	p-value	Fold Change	Location	Family
**CCL2**	0.001	8.4	Extracellular Space	cytokine
**IL1RL1**	0.001	8.3	Plasma Membrane	transmembrane receptor
**CXCL8**	0.000	8.2	Extracellular Space	cytokine
**SERPINE1**	0.002	7.1	Extracellular Space	other
**ICAM1**	0.018	4.6	Plasma Membrane	transmembrane receptor
**STAT3**	0.017	3.7	Nucleus	transcription regulator
**YWHAE**	0.041	2.7	Cytoplasm	other
**CCL3**	0.041	2.6	Extracellular Space	cytokine
**HSPA5**	0.034	2.5	Cytoplasm	enzyme
**HNRNPA1**	0.016	2.2	Nucleus	enzyme
**PABPC1**	0.004	2.1	Cytoplasm	translation regulator
**SELE**	0.023	2.1	Plasma Membrane	transmembrane receptor
**ITGA5**	0.014	2.0	Plasma Membrane	transmembrane receptor
**Hat**			Nucleus	complex
**Hsp90**			Cytoplasm	group

Additional file 3: Table S2

Transcripts affected in FFPE heart tissue samples from meningococcal septic shock patients

Data obtained from a “core analysis “ in IPA (Fig. 7B), (FC ≥ |±2|, p<0.05, BH 1.3 and Z-score |±1|).

Symbol	p-value	Fold Change	Location	Family
**EIF1**	0.032	2.2	Cytoplasm	translation regulator
**FOS**	0.029	2.3	Nucleus	transcription regulator
**EIF2S3**	0.007	2.4	Cytoplasm	translation regulator
**SERPINA1**	0.023	2.5	Extracellular Space	other
**HP**	0.007	3.1	Extracellular Space	peptidase
**C3**	0.046	3.7	Extracellular Space	peptidase
**SOD2**	0.042	4.3	Cytoplasm	enzyme
**SERPINE1**	0.046	5.0	Extracellular Space	other
**SERPINA3**	0.023	5.4	Extracellular Space	other
**43S Translation Preinitiation**			Cytoplasm	complex
**48s**			Cytoplasm	complex
**Eif2**			Cytoplasm	complex

Additional file 3: Table S3.

Transcripts affected in FFPE kidney tissue samples from meningococcal septic shock patients

Data obtained from a “core analysis “ in IPA (Fig. 7C), (FC ≥ |±2|, p<0.05, BH 1.3 and Z-score |±1|).

Symbol	p-value	Fold Change	Location	Family
**APOA5**	0,000	-2.4	Extracellular Space	transporter
**HDL**			Plasma Membrane	complex
**LDL**			Plasma Membrane	complex

Additional file 3: Table S4.

Transcripts affected in FFPE liver tissue samples from meningococcal septic shock patients

Data obtained from a “core analysis “ in IPA (Fig. 7D), (FC ≥ |±2|, $p<0.05$, BH 1.3 and Z-score |±1|).

Symbol	p-value	Fold Change	Location	Family
**COX6C**	0.022	4.0	**Cytoplasm**	**enzyme**
**COX8A**	0.021	3.0	**Cytoplasm**	**enzyme**
**ATP5F1C**	0.042	2.1	**Cytoplasm**	**transporter**
**cytochrome-c oxidase**			**Cytoplasm**	**complex**
**F1 ATPase**			**Cytoplasm**	**complex**

Additional file 3: Table S5.

Transcripts affected in FFPE spleen tissue samples from meningococcal septic shock patients

Data obtained from a “core analysis “ in IPA (Fig. 7E) (FC ≥ |±2|, $p<0.05$, BH 1.3 and no Z-score limit)
